# Higher Serum Angiotensinogen Is an Indicator of IgA Vasculitis with Nephritis Revealed by Comparative Proteomes Analysis

**DOI:** 10.1371/journal.pone.0130536

**Published:** 2015-06-22

**Authors:** Xuelian He, Wei Yin, Yan Ding, Shu-jian Cui, Jiangwei Luan, Peiwei Zhao, Xin Yue, Chunhua Yu, Xiaohui Laing, YuLan Zhao

**Affiliations:** 1 Clinical research center, Wuhan Children’s Hospital, No. 100 Hongkong Rd,Wuhan, China; 2 Department of Rheumatology, Wuhan Children’s Hospital, No. 100 Hongkong Rd,Wuhan, China; 3 Key Laboratory of Crop Genetics and Physiology of Jiangsu Province, College of Bioscience and Biotechnology, Yangzhou University, Yangzhou 225009, China; 4 Department of Nephrology, Wuhan Children’s Hospital, No. 100 Hongkong Rd,Wuhan, China; 5 School of Public Health, Wuhan University, Wuhan, China; 6 Institute of Advanced Interdisciplinary Research, East China Normal University, Shanghai, China; Deutsches Krebsforschungszentrum, GERMANY

## Abstract

IgA vasculitis (IgAV), previously named as Henoch–Schönlein purpura, is the most common systematic vasculitis with unknown etiology. Lack of appropriate study system and/or animal model limits the understanding of its molecular pathogenesis and hinders the identification of targets for rational therapy, especially for its long-term complication, IgAV nephritis (IgAVN). In this study, we applied comparative analysis of serum proteomes to obtain an insight about disease pathogenesis. This study has utilized high sensitivity nanoscale ultra performance liquid chromatography-mass spectrometry (nanoLC-MS/MS) to investigate the alterations in serum proteomic profiles in patients with IgAV (n=6), IgAVN (n=6) and healthy subjects (n=7). The differentially expressed proteins were subjected to functional pathway analysis by PANTHER and DAVID software. We identified 107 differentially expressed proteins among three different groups, and functional analysis suggested that, in addition to earlier reported pathways, such as acute phase response, immune response, complement and blood coagulation pathways, hemostasis and Wnt signaling pathway were probably involved in pathogenesis of IgAV. A few differentially abundant proteins identified, such as C4a, serum amyloid A, angiotensinogen, and kininogen 1, were further validated by ELISA. More importantly, we found that angiotensinogen concentration is correlated with IgAVN and could be used as a potential marker for the progression of IgAV. This is the first report of analyzing the proteomic alterations in IgAV patients and the differentially proteins identified in this study may enhance understanding of the pathology of IgAV and a few of them may be used to monitor disease progression.

## Introduction

Henoch—Schönlein purpura (IgAV) is the most common systematic vasculitis disease in childhood, characterized by the presence of immunoglobulin A1 (IgA1) dominant immune deposits in the small vessels. It occurs most commonly in the autumn and winter with an incidence of 10–20 per 100,000 populations [1–2]. Renal involvement is the most serious long-term complication, and the signs of renal involvement include asymptomatic microhematuria and/or mild proteinuria to overt IgAV nephritis (IgAVN) [3]. IgAVN, occurring in approximately 30% pediatric patients within 4–6 weeks of the initial presentation [4], and severe IgAVN can be associated with decreased renal function, hypertension, hypoalbuminemia, and long-term renal sequelae. Current treatment for IgAVN, including steroids and immunosuppressive drugs, are mainly based on results from studies on IgA nephritis (IgAN) [5]. A better understanding of the pathophysiology of IgAV and the progression to chronic kidney disease is required for better treatment to be achieved. However, as there is no unified system or animal model applicable to research, the study of IgAV and IgAVN has proved challenging.

In the present study, we have performed a comprehensive proteomic analysis of serums from patients suffering IgAV and IgAVN using a high sensitivity NanoLC-MS/MS (nanoflow liquid chromatography interfaced with a linearion trap spectrometer), and compared with healthy controls. We aimed to identify proteins differentially expressed among IgAV, IgAVN and healthy controls. To our knowledge, this is the first report of proteomic analysis in IgAV and IgAVN patients and our results would help reveal the underlying molecular mechanism of disease pathogenesis.

## Materials and Methods

The study protocol was approved by the Institutional Review Board (IRB) of Wuhan Children’s Hospital. We informed the parents of each subject that we would anonymously use the medical reports, blood samples, and related clinical parameters in our study, and we obtained verbal consent but not written consent as the data were anonymously analyzed and reported. Our IRB approved this consent procedure.

### Patient selection and study design

The active diagnosis of IgAV was following the criteria proposed by the European League against Rheumatism/the Paediatric Rheumatology European Society (EULAR/PReS) in 2005 [6]. IgAVN was diagnosed if the patients had hematuria (≥5 red blood cells/hpf) and/or proteinuria (>300 mg/24 h) and/or nephritic syndrome (>3.5 g/day proteinuria with serum albumin (<25g/L). After approvaled by the hospital’s medical ethical committee and informed consent was obtained, 12 patients, including 6 active IgAV patients, 6 IgAVN, and 7 age- and gender-matched health controls, were enrolled in the study. The disease severity was assessed by clinical system according to the involvement of joint, gastro intestine, and kidney. The patients were divided into two groups based on clinical presentation: high clinical score (HCS) group if clinical score ≥4 and low clinical score (LCS) group if clinical score <4. All IgAV and IgAVN patients had a minimum of 6-month follow-up and had no other immunological diseases. We also included another 63 patients (35 IgAV and 28 IgAVN) and 24 healthy controls for validation. In addition, another consecutive 102 patients with active IgAV were collected to investigate the biomarker for predicting the progression of IgAV, and we followed up these patients at least 6 months.

Serum from subjects were collected at the next day of admission and before steroid or other immunosuppressive treatment, and serum from IgAVN patients were obtained when clinical presentations as hematuria and/or proteinuria and/or nephritic syndrome, were detected.

### Sample processing

Three separate pools, health controls (n = 7), active IgAV (n = 6), and IgAVN (n = 6), were created. To avoid the individual difference, the serum samples in the same group were mixed at same volume (100μL) with similar protein concentrations. The albumin/IgG in the serum was removed and the remaining proteins were quantified. Protein in-solution digestion and strong cation exchange (SCX)-200 μg proteins were digested, respectively. First, proteins were treated with 10 mM dithiothreitol (DTT) and then carboxamidomethylated in 55 mM iodoacetamide. Next, the protein mixtures were diluted with deionized water and digested overnight in 50 mM NH_4_HCO_3_ with sequencing grade modified bovine trypsin (Roche Applied Science). On the next day, a further four-hour digestion was carried out by adding the same amount of trypsin to the mixture. Then the typtic peptide mixture was diluted 10-fold with deionization water/formic acid (FA) (pH 3.0) and loaded to a SCX chromatography column (Applied Biosystems). The peptide mixture was then fractionated into 10 subgroups by SCX chromatography using ammonium acetate. Each SCX fraction was desalted using reverse phase (RP) chromatography.

### NanoLC-MS/MS

The tryptic digests were then loaded onto a RP trap column (C18, 5μm, 300 Å, 300 mm id × 5 mm,Waters) for enrichment at a flow rate of 10μL/min. The trap column was sequentially connected in-line with an analytical column (75μm × 150 mm C18, Waters) and the peptide mixtures were eluted into SYNAPT G2 (Waters) at a flow rate of 200 nL/min. NanoUPLC(Waters) was used to deliver mobile phases A (0.5% acetic acid in water) and B (0.5% acetic acid in ACN) at a linear gradient from 5% B to 50% B within 60 min, along with a gradient from 50% B to 90% B within 30 min and then 90% B for 15 min. A spray voltage of 3000 V was applied to a nanospray emitter (New Objective) connected at the end of the analytical column through a stainless union joint (Valco Instrument) to give a steady spray.

### Data base search and analysis

The data were postacquisition lock mass corrected using the doubly charged monoisotopic ion of [Glu1]-fibrinopeptide B. The reference sprayer was d with a frequency of 30 s. Accurate mass LC-MS data were collected in an alternating, low energy, and elevated-energy mode of acquisition. The spectral acquisition time in each mode was 1.2 s. In low energy MS mode, data were collected at constant collision energy of 4 eV. In elevated-energy MS mode, the collision energy was ramped from 15 to 50 eV during each 1.2 s integration. The scan window was set from m/z 100 to 1800. The MSE DATA were searched against the human protein databases (IPI, HUMAN, V3.72) using ProteinLynx Global SERVER (PLGS 2.5) (WATERS). Searching parameters as followings: the Value of Min Fragment Ion Matches per Peptide was 3, the value of Min Fragment Ion Matches per Protein was 7, and the value of Min Peptide Matches per Protein was 1; Trypsin was set as digest reagent, the allowed number of Missed Cleavages was 2; Carbamidomethyl C was set as fixed modification, Oxidation M and Phosphoryl STY were set as variable modifications. The False Positive Rate was less than 4%. The Expression Analyses program with Auto Normalization was employed for quantitation analysis. Relative quantities of the identified proteins were represented by the quotient of the number of MS/MS normalized by total peptides identified in individual group and the summed PLGS scores of proteins [7].

### Protein networks and functional analysis

The differentially expressed proteins between healthy controls and patients, including IgAV and IgAVN patients, as well as those between IgAV and IgAVN, were subjected to functional pathway analysis using PANTHER software, version 7 (http://www.pantherdb.org) [8] and Database for Annotation Visualization, and Integrated Discovery (DAVID) database version 6.7 (http://david.abcc.ncifcrf.gov/home.jsp) [9] for better understanding of the biological context of these proteins and their potential roles and physiological pathway in the pathogenesis of IgAV and IgAVN. A biological process or pathway was considered to be significant if it contained a minimum of three proteins per category featuring score values less than 0.05 after Benjamini-Hochberg correction.

### Serum amyloid A (SAA1), C4a, Angiotensinogen (AGT) and Kininogen 1(KNG1) measurement by ELISA

To follow up the finding by mass spectrometry, serum concentrations of SAA1, C4a, AGT, and KNG1 were determined by ELISA kit (SAA1 from Abcam; AGT, C4a and KNG1 from Uscn life Science) in the validation cohort (63 IgAV and 24 healthy controls). In addition, based on the results and previous studies, AGT was measured in another active 102 IgVA patients in order to investigate whether it could predict the progression of IgAV. The data were presented as mean ± standard deviation (SD). Differences were considered significant for p<0.05. Student t test was used based on the normal distribution of the data. The Perason’s correlation coefficient was used to assess the correlation between different proteins, and the area under the receiver operating characteristic (ROC) curve (AUC) to evaluate the prediction performance of biomarkers on risk of developing IgAVN. All statistical analyses were performed using SPSS v.16.

## Results

### NanoLC-MS/MS analysis

**Fig 1:**
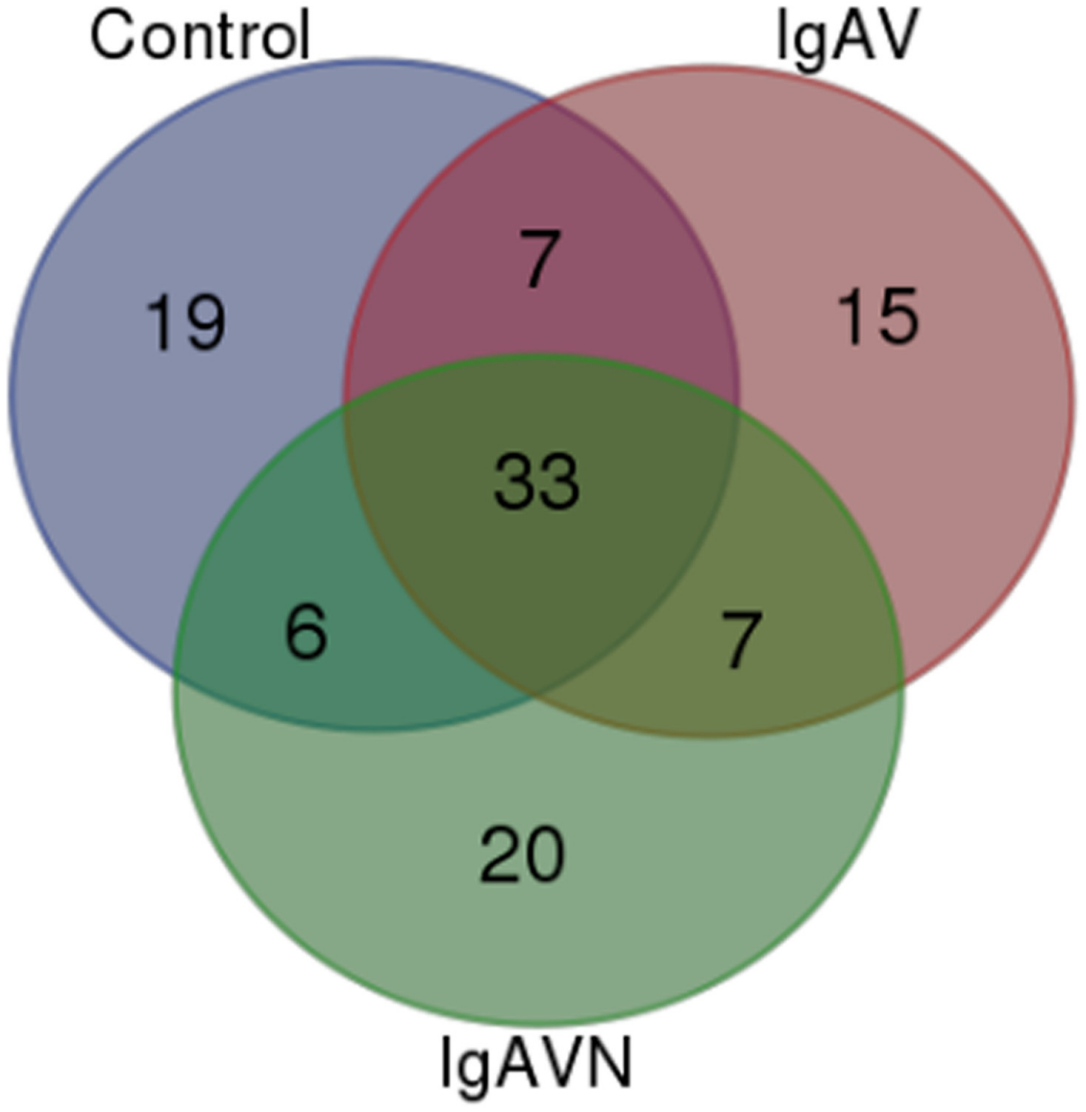
Summary of the NanoLC—MS/MS data of samples. Venn diagram representing the overlap of proteins with at least 4 unique peptides identified among different groups.

The demographic and clinical characteristics of patients for proteomic analysis were presented in Table 1. We investigated the alteration of serum proteome of IgAV and IgAVN patients by analyzing three pools from different conditions using nanoLC-MS/MS approach. There were 4743, 4792 and 4345 peptides were used to identify 263, 266 and 260 proteins in control, IgAV and IgAVN, respectively. As shown in S1 Table, there were 212, 192 and 195 peptides with at least 4 valid peptides in control, IgAV and IgAVN, respectively [10]. As a number of proteins were represented by a few protein fragments, after further analyzing these proteins, we chose representative proteins and identified differentially expressed proteins in three groups. In order to improve the reliability, only proteins with more than 4 unique peptides and/or with 2-fold change were further analyzed. Fig 1 shows the complementarities of proteins with at least 4 valid peptides in three groups: besides the 33 overlapping proteins, there were another 7 proteins common in IgAV and IgAVN groups, and 15 specific for IgAV and 20 specific for IgAVN. The details of proteins were shown in Table 2.

**Table 1:** The demographic and clinical characteristics of children with IgAV or IgAVN.

						Other involvement						
	No. of patient	Age (y)	Sex	Time from onset (days)	Clinical scores	Arthralagias and/or arthritis	Bowel angina and/or Gastrointestinal bleeding	Proteinuria/ hematuria	IgA (g/L)	CRP (mg/L)	C3 (g/L)	C4 (g/L)
	1	6	1	19	4	1	3	0	1.19	3.16	1.06	0.35
Active	2	9	1	16	1	1	0	1	2.20	0.77	0.76	0.22
IgAV	3	5	1	8	3	0	0	0	1.68	0.77	1.13	0.22
without	4	6	2	1	2	2	0	0	2.51	1.85	0.69	0.09
nephritis	5	6	2	3	1	0	1	0	1.66	2.56	0.83	0.20
	6	10	1	20	0	1	3	0	2.54	1.53	1.04	0.17
	1	9	1	15	3	0	0	3/3	4.56	21.20	1.18	0.33
	2	8	1	20	4	0	1	0/3	1.11	1.03	1.02	0.19
Active	3	7	2	6	4	1	0	2/3	1.52	0.77	0.90	0.23
IgAVN	4	5	2	30	3	0	0	3/0	2.65	0.77	1.21	0.26
	5	9	1	7	4	1	0	1/3	7.06	15.20	1.24	0.17
	6	3	1	10	5	0	2	3/2	1.12	11.30	1.26	0.22

Joint: 0 = no symptoms;1 = pain and/or slightly swelling; 2 = pain and/or moderately swelling; 3 = pain and/or severely swelling; GI: 0 = no symptom; 1 = slight pain and/or occult stool blood(OSB) (+); 2 = moderate pain and/or OSB(+2,+3); 3 = severe and/or maelena; Kidney: 0 = no proteinuria; 1 = proteinuria(+) and/or hematuria(+); 2 = proteinuria(2+,3+) and/or hematuria(2+,3+); 3 = proteinuria(>3+) and/or hematuria(>3+).

**Table 2:** List of differentially expressed proteins identified in IgAV and/or IgAVN patients compared to healthy controls.

				PLGS Score^a^			Peptides^b^			
IPI No.	Description	mW (kDa)	pI (pH)	C	IgAV	IgAVN	C	IgAV	IgAVN	Molecular Function
**Proteins common in control, IgAV, IgAVN**										
00292530	ITIH1 Inter alpha trypsin inhibitor heavy chain H1	101.33	6.31	2631.61	2795.74	1330.60	48	60	21	binding, catalytic activity
00892547	C4A component 4A	192.75	6.58	3377.31	4536.46	6322.23	36	84	80	binding, enzyme regulator activity
00400826	CLU clusterin isoform 1	57.80	6.23	1628.70	977.73	1127.35	34	9	17	Apoptosis
00019943	AFM Afamin	69.02	5.54	520.25	838.90	210.25	26	34	11	transfer protein
00305461	ITIH2 Inter alpha trypsin inhibitor heavy chain H2	106.37	6.40	1931.78	4086.38	1406.37	26	53	15	enzyme regulator activity
00607707	HPR Isoform 2 of Haptoglobin related protein	43.03	6.46	2559.64	2683.53	1251.63	25	27	10	binding, catalytic activity
00166729	alpha 2 glycoprotein 1 zinc	34.24	5.64	798.28	1246.33	1955.65	21	24	44	receptor activity
00479708	IGHM protein	68.57	6.89	1117.21	509.41	375.23	19	5	5	antigen binding
00922744	C4B Complement protein C4B	38.11	8.59	1992.75	968.88	1352.70	18	10	18	binding, enzyme regulator activity
00032179	SERPINC1 Antithrombin III	52.66	6.06	848.34	337.27	686.34	15	7	18	enzyme regulator activity
00386524	IGHA1 IGHV3OR16	53.50	6.21	1894.80	3544.99	2121.82	15	34	21	antigen binding
00339225	FN1 Isoform 5 of Fibronectin	243.16	5.36	1051.12	1004.38	1081.32	12	11	28	binding
00885076	IGLC2 IGLV2 14	24.84	8.62	1011.23	2045.85	1063.10	12	27	13	antigen binding
00215894	KNG1 Isoform LMW of Kininogen 1	47.85	6.26	829.53	2549.45	1639.44	12	31	25	enzyme regulator
00382938	IGLV4 3 IGLV4 3 protein	25.96	6.32	988.11	1983.62	1021.91	11	26	17	N/A
00736763	SERPINA2	47.86	7.88	214.86	170.40	159.43	10	5	4	enzyme regulator
00440577	IGKV2 24	26.23	8.40	916.13	1532.96	403.35	10	20	6	N/A
00021842	APOE Apolipoprotein E	36.13	5.48	919.48	1800.03	1033.35	9	21	24	binding
00793626	CP 22 kDa protein	22.06	4.78	359.79	259.67	206.77	7	2	2	Transporter activity
00789376	KNG1 KNG1 protein	33.06	6.26	253.77	1098.15	1328.57	7	19	26	enzyme regulator
00796636	HBB Hemoglobin Fragment	11.50	5.90	349.40	1120.29	609.62	6	17	10	oxygen transport
00853068	HBA2 HBA1 Alpha 2 globin	15.27	9.18	531.43	138.66	370.98	5	3	7	oxygen transport
00022429	ORM1 Alpha 1 acid glycoprotein 1	23.50	4.74	366.59	464.51	951.10	4	3	11	transport
00032220	AGT Angiotensinogen	53.12	5.85	1210.17	1086.63	620.02	4	13	15	enzyme regulator activity
00642632	C7 protein	11.35	8.48	272.72	537.35	757.36	3	6	11	Complement
00852577	C1 segment protein	11.39	7.99	214.90	580.46	216.43	3	9	3	N/A
00853641	HBE1 Putative uncharacterized protein HBE1	9463	9.7189	135.579	117.82	228.8153	2	2	4	oxygen binding
00178926	IGJ immunoglobulin J chain	18.09	4.91	168.39	97.56	115.27	2	4	1	antigen binding
00657670	Apolipoprotein C III variant 1	12.81	8.72	699.19	925.38	759.11	2	6	5	binding, enzyme regulator activity
00022432	TTR Transthyretin	15.88	5.40	153.21	737.29	99.50	2	7	2	transport activity
00798430	TF 12 kDa protein	12.04	9.11	463.98	154.49	113.90	10	2	3	transport activity
00658130	IGL protein	25.01	7.98	333.72	1256.64	808.11	2	15	11	N/A
00761125	IGKC protein	25.66	8.36	371.29	1026.87	384.40	1	7	2	antigen binding
**Proteins shared between controls and IgAV**										
00742696	GC vitamin D binding protein precursor	52.88	5.15	889.85	269.64		30	5		Transport
00867588	FN1 Isoform 13 of Fibronectin	249.15	5.25	2165.21	2230.92		23	25		binding
00555812	GC Vitamin D binding protein	52.93	5.24	864.50	271.52		22	10		Transport
00745089	A1BG alpha 1B glycoprotein precursor	54.22	5.48	1282.17	1049.89		17	12		Cellular component
00793848	CLU 54 kDa protein	53.48	6.52	284.04	152.45		9	6		Apoptosis
00291262	CLU Clusterin	52.46	5.84	694.15	172.28		6	3		Apoptosis
00479116	CPN2 Carboxypeptidase N subunit 2	60.58	5.57	262.70	208.80		3	4		receptor activity
**Proteins shared between IgAV and IgAVN**										
00022395	C9 component C9	63.13	5.27		457.48	1334.26		12	38	receptor activity, transport activity
00797097	KNG1 17 kDa protein	17.34	4.63		718.21	249.91		14	3	enzyme regulator
00298971	VTN Vitronectin	54.27	5.43		323.85	660.93		5	22	binding
00019399	SAA4 Serum amyloid A 4	14.80	9.52		242.42	252.06		8	5	transport activity
00218192	ITIH4 Isoform 2	101.15	6.20		503.26	1591.03		3	14	binding, catalytic activity
00855916	Transthyretin	20.19	4.97		334.40	99.50		3	4	transport activity
00021857	APOC3 Apolipoprotein C III	10.85	5.05		253.25	567.25		1	8	binding, transport activity
**Proteins shared between Controls and IgAVN**										
00339224	FN1 Isoform 4 of Fibronectin	222.80	5.30	2010.77		2059.00	22		32	binding
00339226	FN1 Isoform 6 of Fibronectin	240.32	5.31	2124.12		1036.02	21		15	binding
00884981	PZP Isoform 2 of Pregnancy zone protein	140.28	5.86	689.11		437.47	14		5	binding,catalytic activity
00025426	PZP Isoform 1 of Pregnancy zone protein	163.73	5.93	719.08		494.93	7		5	binding,catalytic activity,enzyme regulator activity
00878729	A2M 19 kDa protein	18.71	6.09	791.78		621.68	6		4	enzyme regulator
00514475	APOL1 Isoform 1 of Apolipoprotein L1	43.95	5.47	106.02		170.72	5		5	transport activity
**Proteins only in Controls**										
00382606	F7 Factor VII active site	75.50	6.59	1050.42			19			coagulation
00448938	IGHG1 IGHG1 protein	51.36	8.44	978.12			18			antigen binding
00411462	FN1 Isoform 2 of Fibronectin	71.90	6.54	314.85			17			binding
00290283	MASP1 isoform 2 precursor	81.81	4.82	258.21			12			catalytic activity
00219561	NLRP14 NACHT LRR and PYD domains containing protein 14	124.65	6.16	443.78			15			binding, transcription
00175193	KIF4B kinesin	139.95	5.79	351.96			15			structural molecular activity
00017891	APC2 Isoform 2	213.67	9.68	529.71			29			Signal transduction
00556059	KIF4A Isoform 2 of Chromosome associated kinesin KIF4A	128.38	5.81	262.20			11			catalytic activity, structural molecular activity
00914853	LRRFIP1 leucine rich repeat in FLII interacting protein 1 isoform 2	44.88	5.34	196.62			9			binding
00641877	WNT2B Protein Wnt	33.92	8.88	82.65			8			binding
00888398	RNF187 Protein RNF187	14.61	5.82	139.03			7			binding, catalytic activity
00795830	AHSG 29 kDa protein	28.52	4.55	174.02			7			enzyme regulator
00550640	IGHG4 IGHG4 protein	51.95	7.84	550.43			7			antigen binding
00005686	LIPG Isoform 1 of Endothelial lipase	56.76	7.82	211.33			6			catalytic activity
00020986	LUM Lumican	38.40	6.16	256.79			5			receptor activity
00013698	ASAH1 Acid ceramidase	44.62	7.52	101.87			5			catalytic activity
00399007	IGHG2	46.03	7.42	406.99			5			antigen binding
00002919	DIRAS1 GTP binding protein Di Ras1	22.31	8.94	133.72			4			binding,catalytic activity
00023019	SHBG Isoform 1	43.75	6.24	133.43			4			catalytic activity
**Proteins only in IgAV**										
00844578	DHX9 ATP dependent RNA helicase A	140.87	6.39		338.22			14		binding
00012505	TMPRSS13 Isoform 3 of Transmembrane protease serine 13	57.59	8.55		213.48			12		binding,catalytic activity, enzyme regulator activity
00015175	WNT2 Protein Wnt 2	40.39	8.65		235.70			8		binding
00477357	PLD5 Isoform 3 of Inactive phospholipase D5	37.71	9.56		234.25			8		catalytic activity
00549916	UBXN11 Isoform 7 of UBX domain containing protein 11	32.88	4.78		137.62			9		binding
00019580	PLG Plasminogen	90.51	6.91		242.39			8		catalytic activity
00158144	SYCE2 Synaptonemal complex central element protein 2	24.67	5.47		167.73			6		enzyme regulator
00218732	PON1 Serum paraoxonase arylesterase 1	39.72	4.92		193.95			5		catalytic activity
00552578	SAA1 SAA2 Serum amyloid A protein	13.52	6.35		97.14			5		transport activity
00015388	PAFAH2 Platelet activating factor acetylhydrolase 2 cytoplasmic	44.01	6.44		142.13			4		catalytic activity
00914948	APOL1 apolipoprotein L1 isoform c precursor	42.13	5.46		218.47			4		transport activity
00914985	Epididymis luminal protein 180 Fragment	13.28	5.03		137.08			4		N/A
00382500	Ig heavy chain V III region GAL	12.72	8.81		109.26			4		complement activation
00385985	Ig lambda chain V III region LOI	11.93	4.76		183.53			4		complement activation
00290444	PPP1R15A Protein phosphatase 1 regulatory subunit 15A	73.43	4.36		199.06			4		binding
**Proteins only in IgAVN**										
00296421	EHBP1L1 EH domain binding protein 1 like protein 1	161.76	4.60			339.89			23	binding, structural molecular activity
00829853	AKAP13 Isoform 6 of A kinase anchor protein	49.02	9.19			148.13			10	kinase activity
00026314	GSN Isoform 1 of Gelsolin	85.64	5.84			361.77			9	binding, structural molecular activity
00020091	ORM2 Alpha 1 acid glycoprotein 2	23.59	4.85			269.93			8	transport
00913983	SYN3 synapsin III isoform IIIg	63.18	9.70			220.03			8	ATP binding
00876950	ITIH3 Isoform 2 of Inter alpha trypsin inhibitor heavy chain H3	99.27	5.42			339.90			6	binding, enzyme regulator activity
00218074	FAM9C Protein FAM9C	19.20	4.96			92.67			6	N/A
00023673	LGALS3BP Galectin 3 binding protein	65.29	4.94			196.80			6	catalytic activity, receptor activity
00873416	ITIH3 Putative uncharacterized protein ITIH3	75.03	5.49			288.37			5	catalytic activity
00644018	A1BG 41 kDa protein	40.69	5.40			511.56			5	receptor activity
00031074	ELAVL3	25.63	10.33			191.78			5	binding
00011694	PRSS1 Trypsin 1	26.54	6.07			162.64			4	catalytic activity,binding
00646773	GSN Isoform 2 of Gelsolin	80.59	5.47			362.38			4	binding, structural molecular activity
00029437	KIF9 Isoform 1 of Kinesin like protein KIF9	89.96	6.61			305.86			4	catalyticatalytic activity
00797356	CRBN 11 kDa protein	10.74	9.23			111.18			4	enzyme activity
00884192	GPX4 glutathione peroxidase 4 isoform C precursor	27.03	10.39			236.07			4	catalytic activity
00377087	GSN Gelsolin	20.77	4.49			126.74			4	binding
00003951	LAMA3 15 kDa protein	14.92	9.87			108.92			4	structural molecule activity
00394924	TCF23 Transcription factor 23	23.29	11.72			91.44			4	transcription
00747654	TTN	32.55	5.30			243.44			4	N/A

^a^) The scores are summed PLGS scores of peptides of proteins.

^b^) The number of peptides of proteins identified.

### Modulation of physiological pathway in IgAV and IgAVN studied by functional pathway analysis

**Fig 2:**
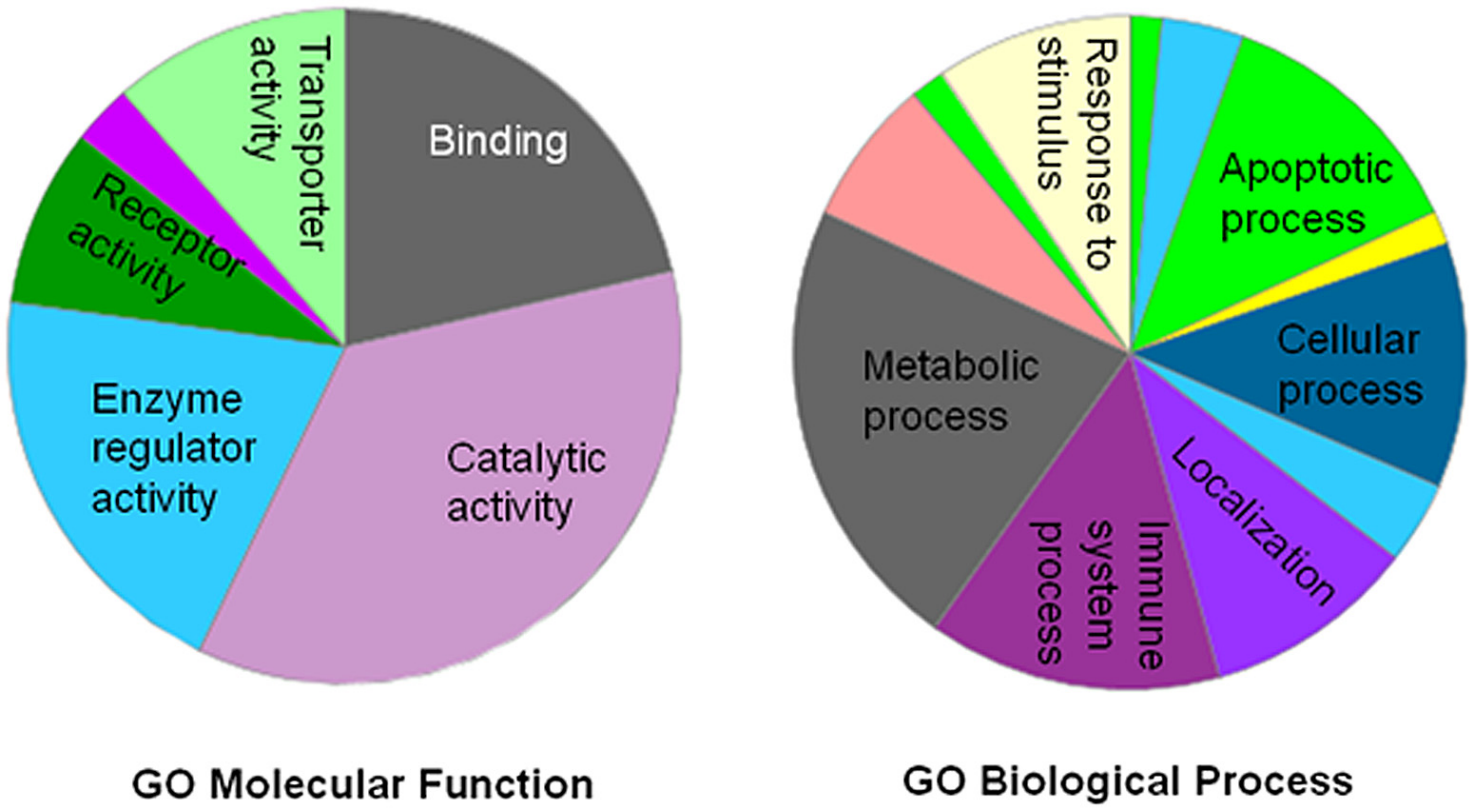
GO molecular function and biological process associated with the differentially expressed proteins identified in IgAV patients. Pie chart represents biological process and molecular obtained in PANTHER analysis.

To further understand the molecular and biological functions of these identified proteins, PANTHER classification system was used and these proteins were mainly classified into metabolic process (21.4%), cellular process (14.3%), immune system process (10.3%), localization (10.3%), response to stimulus (8.7%), and biological regulation (7.9%) (Fig 2). The majority of the identified proteins belonged to 6 major GO molecular functions: catalytic activity (35.3%), binding (23.5%), enzyme regulator activity (11.8%), receptor activity (10.3%), transporter activity (8.8%), and structural molecule activity (7.4%) (Fig 2). In addition, protein classification revealed that a number of proteins were involved in acute phase, defense and immunological responses, such as pregnancy zone protein, zinc-alpha-2-glycoprotein, alpha-1B-glycoprotein, complement 9, galectin-3-binding protein, AHSG, transferring, SAA4, and SAA1. Besides characterize the molecular and biological functions of their proteins, we also used DAVID software to analyze the pathways modulated by these differentially expressed proteins: KEGG category revealed complement and coagulation cascades (p = 6.8E-6, 8.6%) and ECM-receptor interaction (0.046, 4.3%), and blood coagulation pathway was identified in PATHER category (1.1E-5, 8.6%), while Reactome category only revealed homeostasis involved (p = 0.00017, 11.4%). Besides blood coagulation pathway, PATHER also identified Wnt signaling pathway, in which Wnt2, Wnt2B, and adenomatous polyposis coli protein 2 (APC2) were involved. Collectively, these observations suggest that, in addition earlier reported complement and blood coagulation pathways, homeostasis, and Wnt signaling pathways may play previously unsuspected roles in IgAV pathogenesis.

### Validation of differential proteins using immunoassays

**Fig 3:**
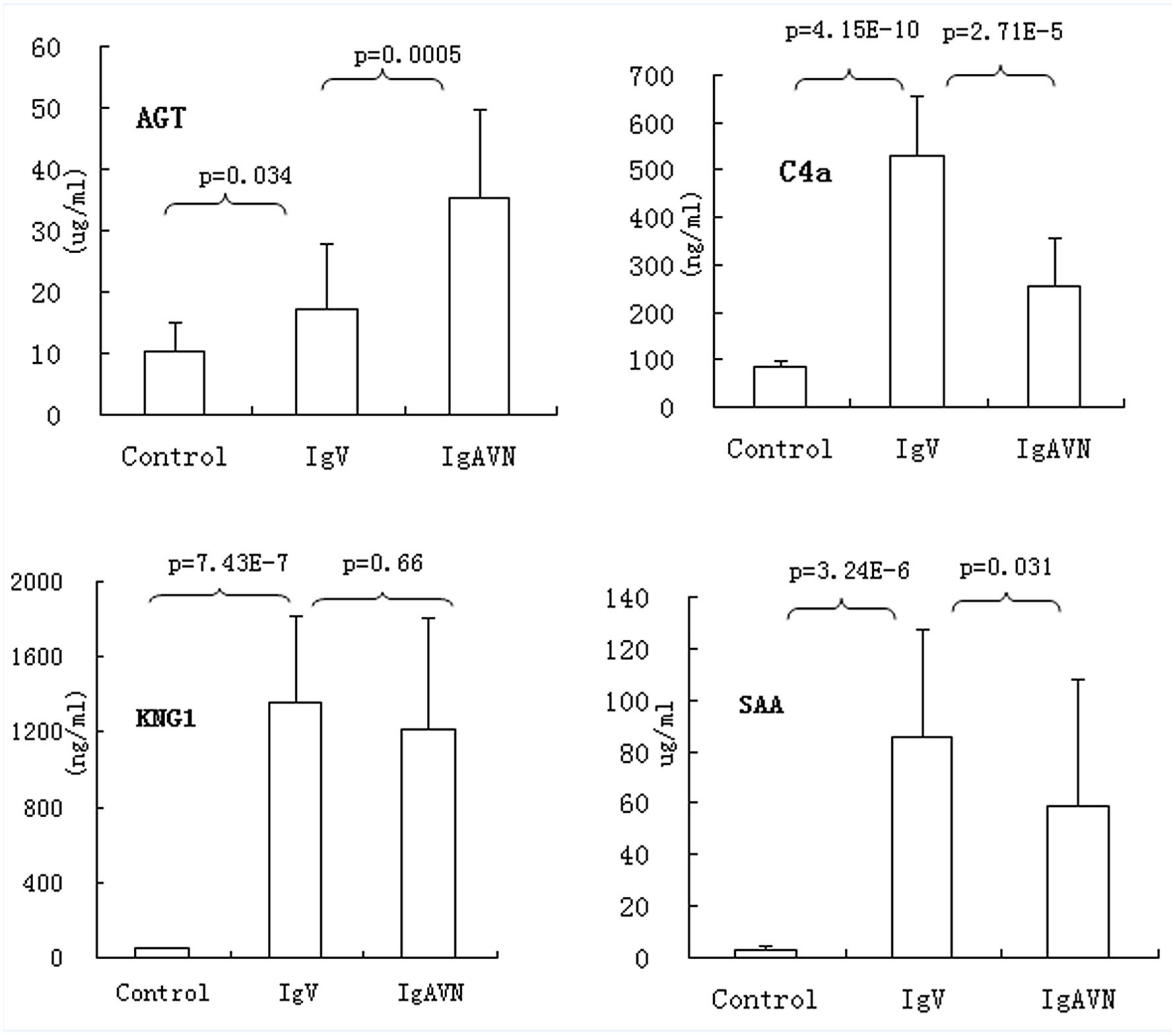
The levels of serum AGT, C4A, SAA1, and KNG1 were measured by ELISA in IgAV, IgAVN and healthy controls (Con). Data represents the mean±SD. The expression levels were compared between different groups, IgAV(35), IgAVN (28), and Con(24) using t test.

In order to validate the results of proteomic analysis, we chose four proteins (SAA1, C4A, AGT, and KNG1) and measured their serum levels by using ELISA kits in the validation cohort, including 35 IgAV, 28 IgAVN, and 24 healthy controls. Consistent with the proteomic results, all four proteins were significantly high in IgAV and/or IgAVN patients (p<0.05). When comparing IgAV with IgAVN, IgAVN had prominently high AGT (p = 0.0005) but lower SAA1 and C4A (p = 0.031, p = 2.71E-5, respectively), and slightly lower KNG1 (p = 0.66) (Fig 3). SAA1 is an acute phase proteins, and it is not surprising that it was increased in patients with active IgAV. Moreover, we also investigated that SAA1 levels were positively correlated with C-reactive protein (CRP) [11], the most commonly used acute phase protein in clinical practice. C4A and KNG1 were not correlated with CRP, and the elevation in these serum proteins could not only due to systemic acute phase reaction but also contribute to the pathogenesis of IgAV. Among the four proteins, AGT is the only protein whose serum level was increased in IgAVN. AGT is the precursor of angiotensin I, which is further converted by ACE into angiotensin II, the key mediator of the renin-angiotensin system pathway. Therapy with ACE inhibitor has been shown to be beneficial in patients with IgAVN, and urine AGT levels were reported to be related to renal involvement of IgAV and may monitor the progression of IgAV. Thus, it is reasonable that serum AGT level is higher in IgAVN compared to that in IgAV in our study. It is worthy to further investigate the predictive role of serum AGT on the progression to IgAVN. For this propose, we collected samples during active phase from 102 patients with IgAV and followed up at least 6-months, among these patients, 16 missed follow-up, and 19 developed into IgAVN. After ELISA analysis, significantly higher AGT levels during active IgAV phase were found in patients who developed to IgAVN (19 cases) than patients who recovered (67 cases) (p<0.0001), and the AUC was 0.833 (95%CI: 0.74–0.93, p<0.001).

## Discussion

Because of the disease benign and self-limited course of IgAV and the lack of appropriate system or animal model, the underlying mechanism of this disease was still unknown. Serum has attracted considerable interest for the clinical studies, as they contain diversity of proteins released by disease tissues [12–14]. Over the last decade, a few proteomic studies have been reported on vasculitis, such as Kawasaki disease, polyangiitis, anti-neutrophil cytoplasmic antibody-associated vasuclitis, and Wegener's granulomatosis [15–19]. These studies helped to identify excellent biological biomarkers for improving diagnostic accuracy, understanding of pathogenesis, and the discovering of novel therapeutic targets. However, to our knowledge, no proteomic analysis has been reported hitherto to discovery biomarkers and/or described the involvement of serum proteins and related biological pathways in IgAV, the most common type of systemic vasculitis.

In this study, we have identified a number of differentially expressed proteins in IgAV and IgAVN and these proteins are involved in the modulation of multiple physiological processes and pathways, including inflammatory and defense responses, acute phase response, complement pathway, blood coagulation and homeostasis. The alterations in acute phase protein expressions were consistent with earlier reports, with AHSG, and TF being decreased and C9, vitronectin, inter-α-trypsin inhibitor heavy H (ITIHs), and SAA1 being increased. The consistency suggested the reliability and enhanced the confidence in this study. Acute phase proteins are involved in defense-related activities by working alone or contribute to inflammatory cascades, such as initiating or sustaining the inflammatory process. More importantly, we found that AGT concentration is correlated with IgAVN and could be used to predict the progress of IgAV, as patients with higher AGT levels during active phase were at high risk of developing into IgAVN. Investigation of the precise biological significance of these acute proteins and their roles in these related pathways in IgAV may provide useful information on the disease pathogenesis.

The role of complement activation in IgAV is controversial. IgAV had been reported to be associated with C2 or deficiency, homozygous null C4 genotypes, and increased C3d levels, furthermore, the membrane attack complex, C5b-9, has been found in skin and renal biopsies [20–21]. The levels of serum C3a and C4a had been shown to be correlated with serum and /or urea creatinine in IgAVN patients and proposed to be used to monitor the progress of disease in these patients [22]. However, inconsistent results were reported [23]. In our study, results reveals altered serum levels of complement components and regulatory proteins, such as C4a, C4b, C9, and vitronectin, were found in IgAV patients compared to healthy controls. In addition, we found C4a levels were decreased in IgAVN than IgAV. Previous studies suggest that complement activation is implicated in tissue damage and is required in IgAVN and IgAN, however, our findings demonstrated that complement activation may be involved in IgAV pathogenesis but serum levels of its components may not be correlated to the severity of the disease.

We have identified a number of proteins regulating peptidase activity, including C4B, transmembrane protease serine 13, ITIHs, AGT, ceruloplasmin, C4A, α-1-antichymotrypsin, α-1-antitrypsin, and A2M. Proteinases and their inhibitors are key regulators of numerous biological pathways that initiate inflammation, coagulation, complement activation, apoptosis, extra-cellular matrix composition and angiogenesis responses [24]. Our proteomic analysis raises the possibility that proteolysis could play a part in the pathogenesis of IgAV, and explain the clinical manifestations, including palpable purpura, pain, and oedema. In addition, non-thrombocytopenic palpable purpura is one of major manifestations and the precise reasons and pathophysiological mechanisms are not clear. Coregulators in blood coagulation cascade, including KNG1, PLG, SERPINC1, have been found to be altered in IgAV patients. Thus, we proposed that inflammation-induced coagulation activation within blood vessels could contribute to the palpable purpura and secondary hyperfibrinolysis results in hematuria and gastrointestinal bleeding. Detailed investigation of functional aspects of these proteins related to coagulation and secondary hyperfibrinolysis might provide interesting insights about these clinical features of IgAV.

Our study showed that Wnt signaling pathway could be involved in the modulation of IgAV pathogenesis, which is due to the alteration of Wnt2, Wnt2B, and APC2. Wnt2 pathway has been suggested to contribute to the protection to pathogen infection and inflammation [25]. As IgAV has been proposed to be trigged by a wide variety of microbial antigens, whether the alteration of Wnt signaling pathway plays a role in the pathogenesis or is just a consequence of this disease need to be established.

Some limitations in our study should be addressed. First, other forms of small vessel vasculitides involving kidney damage such as ANCA-associated vasculitis or cryoglobulinemic vasculitis should be included in order to assess specificity of the findings. Second, the duration of following-up should be longer, as it is possible that some IgAV patients without nephritis could relapse and develop into IgAVN.

## Conclusions

This is the first study to investigate the serum proteome in IgAV and IgAVN patients. We found that AGT concentration is correlated with IgAVN and could be used as a potential marker for the progression of IgAV. In addition, our results suggested earlier reported complement and coagulation pathways could be involved in the pathogenesis, and whether Wnt signaling pathway has a role need to be established. Further investigation of precise biological significance of these identified proteins may provide a better understanding of disease pathogenesis and aid in identification of potential therapeutic targets.

## Supporting Information

S1 Table Proteins identified by nano-LC-MS/MS in controls, IgAV, and IgAVN. Notes: C, control; pI, isoelectric poin; mW, molecular weight.
(DOC)
