# Supplementary material for: Higher Serum Angiotensinogen Is an Indicator of IgA Vasculitis with Nephritis Revealed by Comparative Proteomes Analysis
Source: PLoS One. 2015 Jun 22;10(6):e0130536. doi: 10.1371/journal.pone.0130536 (PMC4476708; doi:10.1371/journal.pone.0130536)
Supplement: S1 Table — Notes: C, control; pI, isoelectric poin; mW, molecular weight. (DOC) [file pone.0130536.s001.doc]

Table S1 List of all proteins identified in three groups.

| **Accession No.** | **Description** | **mW(kDa)** | **pI (pH)** | **PLGS Score** | | | **Peptides** | | |
| --- | --- | --- | --- | --- | --- | --- | --- | --- | --- |
|  |  |  |  | **C** | **IgAV** | **IgAVN** | **C** | **IgAV** | **IgAVN** |
| **Proteins common in control, IgAV, IgAVN** | | | | | | | | | |
| IPI00783987.2 | C3 Complement C3 Fragment | 187.03 | 5.96 | 17696.42 | 17414.45 | 18271.26 | 280 | 294 | 255 |
| IPI00478003.1 | A2M Alpha 2 macroglobulin | 163.17 | 5.97 | 18067.58 | 16764.01 | 19055.64 | 277 | 274 | 268 |
| IPI00887739.2 | LOC100133511 cDNA FLJ57339 highly similar to Complement C3 | 122.52 | 6.49 | 11352.50 | 12769.56 | 10582.41 | 176 | 179 | 147 |
| IPI00022463.1 | TF Serotransferrin | 77.00 | 6.75 | 6404.15 | 6768.53 | 5725.10 | 141 | 152 | 135 |
| IPI00853525.1 | APOA1 Apolipoprotein A1 | 27.89 | 5.73 | 5677.29 | 6065.16 | 4574.32 | 105 | 126 | 78 |
| IPI00021841.1 | APOA1 Apolipoprotein A I | 30.76 | 5.43 | 7703.43 | 8799.58 | 6370.60 | 90 | 98 | 79 |
| IPI00847179.1 | APOA4 apolipoprotein A IV precursor | 45.34 | 5.11 | 4007.69 | 2896.84 | 2302.55 | 75 | 47 | 35 |
| IPI00017601.1 | CP Ceruloplasmin | 122.13 | 5.34 | 2783.49 | 2038.59 | 2744.21 | 74 | 55 | 56 |
| IPI00902590.1 | HP Haptoglobin | 45.18 | 6.12 | 4652.70 | 4111.36 | 4205.08 | 69 | 65 | 73 |
| IPI00304273.2 | APOA4 Apolipoprotein A IV | 45.37 | 5.11 | 3764.11 | 3120.73 | 2411.51 | 68 | 65 | 42 |
| IPI00894122.1 | APOB Putative uncharacterized protein APOB | 92.56 | 7.15 | 1889.56 | 1234.48 | 2177.82 | 66 | 42 | 69 |
| IPI00643525.1 | C4A Putative uncharacterized protein ENSP00000372815 | 192.62 | 6.64 | 4936.48 | 4611.04 | 3468.33 | 57 | 56 | 38 |
| IPI00892604.1 | C4B Complement component C4B Childo blood group 2 | 192.68 | 6.87 | 4187.35 | 4618.71 | 7097.98 | 56 | 61 | 74 |
| IPI00553177.1 | SERPINA1 Isoform 1 of Alpha 1 antitrypsin | 46.71 | 5.24 | 7872.04 | 10413.54 | 8893.05 | 55 | 84 | 70 |
| IPI00654875.1 | C4B Complement C4 B | 192.67 | 6.72 | 5059.20 | 4737.41 | 5497.43 | 54 | 52 | 59 |
| IPI00902867.1 | HPR cDNA FLJ31310 fis clone LIVER1000165 highly similar to Haptoglobin | 31.39 | 8.37 | 3856.20 | 3500.43 | 3746.97 | 53 | 48 | 36 |
| IPI00478493.3 | HP HPR haptoglobin isoform 2 preproprotein | 38.43 | 6.13 | 4540.75 | 3830.92 | 4418.88 | 52 | 43 | 42 |
| Tabl2 S1 Continued | |  |  |  |  |  |  |  |  |
| **Accession No.** | **Description** | **mW(kDa)** | **pI (pH)** | **PLGS Score** | | | **Peptides** | | |
|  |  |  |  | **C** | **IgAV** | **IgAVN** | **C** | **IgAV** | **IgAVN** |
| IPI00032258.4 | C4A Complement C4 A | 192.65 | 6.64 | 4172.65 | 5258.47 | 5129.19 | 51 | 55 | 58 |
| IPI00887154.2 | C4B Complement component 4B | 192.63 | 6.87 | 4093.04 | 4641.75 | 7123.17 | 49 | 53 | 72 |
| IPI00431645.1 | HP HP protein | 31.36 | 8.37 | 3856.20 | 3500.43 | 3746.97 | 49 | 37 | 40 |
| IPI00418163.3 | C4B complement component 4B preproprotein | 192.63 | 6.87 | 4196.24 | 5752.22 | 5919.04 | 48 | 64 | 67 |
| IPI00292530.1 | ITIH1 Inter alpha trypsin inhibitor heavy chain H1 | 101.33 | 6.31 | 2631.61 | 2795.74 | 1330.60 | 48 | 60 | 21 |
| IPI00641737.1 | HPR 47 kDa protein | 46.69 | 6.29 | 5115.74 | 4921.70 | 4844.64 | 46 | 48 | 37 |
| IPI00869004.1 | SERPINA1 Isoform 3 of Alpha 1 antitrypsin | 34.73 | 4.87 | 4152.77 | 4775.01 | 4133.68 | 45 | 56 | 39 |
| IPI00477597.1 | HPR Isoform 1 of Haptoglobin related protein | 38.98 | 6.44 | 2894.33 | 3503.53 | 2009.79 | 45 | 50 | 27 |
| IPI00847635.1 | SERPINA3 Isoform 1 of Alpha 1 antichymotrypsin | 47.62 | 5.18 | 2306.55 | 1744.92 | 2667.19 | 45 | 27 | 39 |
| IPI00889723.1 | C4A complement component 4A preproprotein | 192.66 | 6.64 | 3395.97 | 4628.63 | 5541.19 | 42 | 48 | 62 |
| IPI00739237.1 | LOC653879 similar to complement component 3 | 44.90 | 4.75 | 2521.91 | 2372.42 | 2848.83 | 42 | 40 | 39 |
| IPI00645038.1 | ITIH2 Inter alpha Globulin inhibitor H2 | 105.15 | 6.58 | 1960.49 | 4175.38 | 3269.64 | 41 | 97 | 58 |
| IPI00843913.3 | C4A Complement component 4A | 192.72 | 6.64 | 4183.79 | 4636.46 | 6867.87 | 40 | 50 | 72 |
| IPI00790784.2 | SERPINA1 Isoform 2 of Alpha 1 antitrypsin | 40.24 | 5.12 | 5261.37 | 7631.21 | 7117.56 | 38 | 47 | 51 |
| IPI00556632.3 | FN1 Isoform 12 of Fibronectin | 221.13 | 5.71 | 2031.75 | 2014.06 | 2285.67 | 38 | 40 | 47 |
| IPI00305457.5 | SERPINA1 PRO2275 | 13.09 | 9.58 | 2574.00 | 3755.12 | 3042.44 | 37 | 34 | 29 |
| IPI00892547.1 | C4A Complement component 4A | 192.75 | 6.58 | 3377.31 | 4536.46 | 6322.23 | 36 | 84 | 80 |
| IPI00794184.1 | CP cDNA FLJ37971 fis clone CTONG2009958 highly similar to CERULOPLASMIN | 97.01 | 5.19 | 1489.44 | 592.61 | 2027.57 | 35 | 9 | 29 |
| IPI00386879.1 | IGHA1 IGHV3OR16 13 cDNA FLJ14473 fis clone MAMMA1001080 highly similar to | 53.05 | 6.45 | 3112.40 | 4352.97 | 2436.41 | 33 | 53 | 32 |
| IPI00400826.1 | CLU clusterin isoform 1 | 57.80 | 6.23 | 1628.70 | 977.73 | 1127.35 | 34 | 9 | 17 |
| Tabl2 S1 Continued | |  |  |  |  |  |  |  |  |
| **Accession No.** | **Description** | **mW(kDa)** | **pI (pH)** | **PLGS Score** | | | **Peptides** | | |
|  |  |  |  | **C** | **IgAV** | **IgAVN** | **C** | **IgAV** | **IgAVN** |
| IPI00423462.5 | IGHA1 IGHV3OR16 13 Putative uncharacterized protein DKFZp686K18196 Fragment | 56.39 | 6.50 | 3018.70 | 4699.81 | 2928.13 | 31 | 49 | 29 |
| IPI00795633.1 | CLU cDNA FLJ57622 highly similar to Clusterin | 52.33 | 5.93 | 1568.73 | 606.37 | 1085.27 | 31 | 12 | 17 |
| IPI00305461.2 | ITIH2 Inter alpha trypsin inhibitor heavy chain H2 | 106.37 | 6.40 | 1931.78 | 4086.38 | 1406.37 | 26 | 53 | 15 |
| IPI00922213.1 | FN1 fibronectin 1 isoform 6 preproprotein | 239.48 | 5.50 | 2239.51 | 2306.20 | 2210.21 | 26 | 45 | 31 |
| IPI00019943.1 | AFM Afamin | 69.02 | 5.54 | 520.25 | 838.90 | 210.25 | 26 | 34 | 11 |
| IPI00607707.1 | HPR Isoform 2 of Haptoglobin related protein | 43.03 | 6.46 | 2559.64 | 2683.53 | 1251.63 | 25 | 27 | 10 |
| IPI00022488.1 | HPX Hemopexin | 51.64 | 6.57 | 612.35 | 819.12 | 1133.24 | 25 | 18 | 17 |
| IPI00022895.7 | A1BG Alpha 1B glycoprotein | 54.24 | 5.49 | 1458.38 | 1153.46 | 1133.04 | 24 | 22 | 16 |
| IPI00449920.1 | IGHA1 IGHV3OR16 13 cDNA FLJ90170 fis clone MAMMA1000370 highly similar to | 53.19 | 6.06 | 3079.345 | 4573.222 | 1759.325 | 23 | 41 | 18 |
| IPI00744561.1 | IGHA1 IGHV3OR16 13 IGHA1 protein | 53.36 | 7.68 | 2327.00 | 3610.82 | 2157.52 | 23 | 38 | 21 |
| IPI00550991.3 | SERPINA3 cDNA FLJ35730 fis clone TESTI2003131 highly similar to ALPHA 1 AN | 50.57 | 5.28 | 2317.15 | 2896.69 | 3819.47 | 23 | 35 | 19 |
| IPI00647704.1 | IGHA1 IGHV3OR16 13 cDNA FLJ41552 fis clone COLON2004478 highly similar to | 53.29 | 6.06 | 2928.50 | 3654.36 | 2577.16 | 23 | 32 | 22 |
| IPI00644497.4 | IGHA1 IGHV3OR16 13 Conserved hypothetical protein | 31.78 | 5.31 | 2918.74 | 2413.23 | 1132.05 | 29 | 23 | 10 |
| IPI00855777.1 | FN1 Isoform 14 of Fibronectin | 249.23 | 5.55 | 2208.38 | 2282.24 | 1709.81 | 23 | 25 | 18 |
| IPI00166866.3 | IGHA1 IGHV3OR16 13 | 53.34 | 6.25 | 2286.01 | 3522.17 | 2332.27 | 21 | 38 | 22 |
| IPI00423460.3 | IGHA1 IGHV3OR16 13 Putative uncharacterized protein DKFZp686G21220 Fragment | 54.35 | 6.15 | 2298.88 | 4300.37 | 1867.49 | 22 | 36 | 16 |
| Tabl2 S1 Continued | |  |  |  |  |  |  |  |  |
| **Accession No.** | **Description** | **mW(kDa)** | **pI (pH)** | **PLGS Score** | | | **Peptides** | | |
|  |  |  |  | **C** | **IgAV** | **IgAVN** | **C** | **IgAV** | **IgAVN** |
| IPI00166729.4 | AZGP1 alpha 2 glycoprotein 1 zinc | 34.24 | 5.64 | 798.28 | 1246.33 | 1955.65 | 21 | 24 | 44 |
| IPI00784950.1 | LOC100126583 Putative uncharacterized protein DKFZp686L19235 | 51.61 | 5.39 | 2194.19 | 2414.92 | 1284.62 | 21 | 20 | 12 |
| IPI00021854.1 | APOA2 Apolipoprotein A II | 11.17 | 6.64 | 1275.79 | 982.95 | 1166.98 | 21 | 16 | 16 |
| IPI00061977.1 | IGHA1 IGHV3OR16 13 IGHA1 protein | 54.12 | 6.21 | 1452.58 | 3896.72 | 2141.14 | 20 | 40 | 19 |
| IPI00430842.3 | IGHA1 IGHV3OR16 13 IGHA1 protein | 52.83 | 6.74 | 2301.28 | 3612.33 | 2245.25 | 20 | 33 | 22 |
| IPI00383164.1 | IGHA1 IGHV3OR16 13 SNC66 protein | 53.63 | 6.21 | 2032.58 | 3043.19 | 1648.83 | 19 | 30 | 12 |
| IPI00784830.1 | LOC100126583 cDNA FLJ41981 fis clone SMINT2011888 highly similar to Protei | 52.84 | 5.52 | 1818.58 | 2373.01 | 1367.44 | 19 | 19 | 11 |
| IPI00549291.4 | IGHM IGHM protein | 66.14 | 6.53 | 1083.92 | 899.89 | 932.63 | 19 | 9 | 9 |
| IPI00479708.5 | IGHM IGHM protein | 68.57 | 6.89 | 1117.21 | 509.41 | 375.23 | 19 | 5 | 5 |
| IPI00797833.3 | KNG1 Kininogen 1 | 47.87 | 6.26 | 822.37 | 1623.05 | 1547.07 | 18 | 24 | 19 |
| IPI00922744.1 | C4B Complement protein C4B frameshift mutant Fragment | 38.11 | 8.59 | 1992.75 | 968.88 | 1352.70 | 18 | 10 | 18 |
| IPI00032328.2 | KNG1 Isoform HMW of Kininogen 1 | 71.91 | 6.34 | 934.08 | 1875.17 | 714.27 | 17 | 25 | 10 |
| IPI00719233.1 | IGHA1 IGHV3OR16 13 IGHA1 protein | 53.12 | 6.78 | 2024.28 | 2906.86 | 2391.76 | 17 | 24 | 21 |
| IPI00473011.3 | HBD Hemoglobin subunit delta | 16.05 | 8.24 | 869.34 | 172.98 | 324.71 | 17 | 2 | 7 |
| IPI00032179.2 | SERPINC1 Antithrombin III variant | 52.66 | 6.06 | 848.34 | 337.27 | 686.34 | 15 | 7 | 18 |
| IPI00154742.6 | IGL IGL protein | 24.78 | 5.91 | 1501.17 | 2092.73 | 1445.23 | 14 | 21 | 15 |
| IPI00910636.1 | cDNA FLJ53848 highly similar to Inter alpha trypsin inhibitor heavy chain | 60.67 | 5.00 | 815.04 | 1084.68 | 2103.55 | 13 | 16 | 43 |
| IPI00386524.3 | IGHA1 IGHV3OR16 13 CDNA FLJ25298 fis clone STM07683 | 53.50 | 6.21 | 1894.80 | 3544.99 | 2121.82 | 15 | 34 | 21 |
| Tabl2 S1 Continued | |  |  |  |  |  |  |  |  |
| **Accession No.** | **Description** | **mW(kDa)** | **pI (pH)** | **PLGS Score** | | | **Peptides** | | |
|  |  |  |  | **C** | **IgAV** | **IgAVN** | **C** | **IgAV** | **IgAVN** |
| IPI00642017.1 | IGHA2 Putative uncharacterized protein DKFZp686C02218 Fragment | 53.74 | 6.64 | 1766.19 | 1477.48 | 2097.94 | 15 | 15 | 15 |
| IPI00426060.3 | IGHA1 IGHV3OR16 13 Putative uncharacterized protein DKFZp686J11235 Fragment | 54.42 | 6.32 | 2272.51 | 3999.47 | 2988.61 | 14 | 38 | 28 |
| IPI00654755.3 | HBB Hemoglobin subunit beta | 15.99 | 6.88 | 1410.22 | 1279.08 | 1166.45 | 14 | 21 | 11 |
| IPI00215894.1 | KNG1 Isoform LMW of Kininogen 1 | 47.85 | 6.26 | 829.53 | 2549.45 | 1639.44 | 12 | 31 | 25 |
| IPI00885076.1 | IGLC2 IGLV2 14 IGLC1 IGLC3 IGLV2 14 protein | 24.84 | 8.62 | 1011.23 | 2045.85 | 1063.10 | 12 | 27 | 13 |
| IPI00894384.1 | IGHA2 Putative uncharacterized protein DKFZp686O16217 Fragment | 54.09 | 6.82 | 527.70 | 976.63 | 348.71 | 12 | 23 | 6 |
| IPI00783993.1 | IGHA2 IGHA2 protein | 51.60 | 6.18 | 1671.76 | 1629.97 | 1624.70 | 12 | 13 | 13 |
| IPI00339225.1 | FN1 Isoform 5 of Fibronectin | 243.16 | 5.36 | 1051.12 | 1004.38 | 1081.32 | 12 | 11 | 28 |
| IPI00382938.3 | IGLV4 3 IGLV4 3 protein | 25.96 | 6.32 | 988.11 | 1983.62 | 1021.91 | 11 | 26 | 17 |
| IPI00479723.3 | FN1 Isoform 10 of Fibronectin | 239.46 | 5.48 | 1049.04 | 1033.66 | 1086.72 | 11 | 11 | 15 |
| IPI00410714.5 | HBA2 HBA1 Hemoglobin subunit alpha | 15.25 | 9.18 | 709.22 | 138.66 | 824.23 | 11 | 5 | 7 |
| IPI00889156.1 | IGKV3 20 IGK protein | 25.82 | 6.15 | 1356.88 | 603.68 | 841.08 | 11 | 3 | 6 |
| IPI00879368.1 | APOE Apolipoprotein E | 24.63 | 5.60 | 419.58 | 779.16 | 550.65 | 10 | 21 | 10 |
| IPI00440577.3 | IGKV2 24 IGKV2 24 protein | 26.23 | 8.40 | 916.13 | 1532.96 | 403.35 | 10 | 20 | 6 |
| IPI00514159.1 | ITIH2 Inter alpha Globulin inhibitor H2 | 26.80 | 9.31 | 383.31 | 1361.70 | 732.62 | 10 | 15 | 11 |
| IPI00791097.2 | ITIH4 cDNA FLJ53871 highly similar to Inter alpha trypsin inhibitor heavy c | 62.12 | 5.21 | 352.35 | 449.02 | 414.26 | 10 | 12 | 7 |
| IPI00550731.2 | Putative uncharacterized protein | 26218 | 8.1 | 818.3958 | 1507.649 | 738.5683 | 10 | 10 | 6 |
| IPI00909649.1 | IGKC IGKC protein | 25.63 | 6.15 | 836.49 | 1070.96 | 1279.84 | 10 | 9 | 11 |
| Tabl2 S1 Continued | |  |  |  |  |  |  |  |  |
| **Accession No.** | **Description** | **mW(kDa)** | **pI (pH)** | **PLGS Score** | | | **Peptides** | | |
|  |  |  |  | **C** | **IgAV** | **IgAVN** | **C** | **IgAV** | **IgAVN** |
| IPI00641229.4 | IGHA2 Ig alpha 2 chain C region | 36.50 | 5.66 | 1187.83 | 942.21 | 1379.16 | 10 | 8 | 12 |
| IPI00736763.2 | SERPINA2 Putative alpha 1 antitrypsin related protein | 47.86 | 7.88 | 214.86 | 170.40 | 159.43 | 10 | 5 | 4 |
| IPI00884107.1 | HBB Beta globin gene from a thalassemia patient | 18.92 | 6.31 | 964.73 | 286.95 | 368.88 | 10 | 3 | 2 |
| IPI00798216.1 | TF 12 kDa protein | 12.04 | 9.11 | 463.98 | 154.49 | 113.90 | 10 | 2 | 3 |
| IPI00021842.1 | APOE Apolipoprotein E | 36.13 | 5.48 | 919.48 | 1800.03 | 1033.35 | 9 | 21 | 24 |
| IPI00294193.4 | ITIH4 Isoform 1 of Inter alpha trypsin inhibitor heavy chain H4 | 103.26 | 6.53 | 1290.82 | 811.43 | 469.21 | 9 | 14 | 6 |
| IPI00785067.1 | IGH IGH protein | 51.96 | 5.86 | 1235.49 | 1644.98 | 1256.68 | 9 | 12 | 10 |
| IPI00789547.1 | A2M 19 kDa protein | 18.75 | 8.85 | 1167.56 | 1255.49 | 1997.79 | 9 | 12 | 12 |
| IPI00796467.1 | TF 11 kDa protein | 10.80 | 8.29 | 611.32 | 471.69 | 176.03 | 9 | 9 | 1 |
| IPI00829626.1 | IGL IGL protein | 24.95 | 5.04 | 1319.55 | 847.23 | 348.04 | 12 | 10 | 3 |
| IPI00829640.1 | IGL IGL protein | 24.84 | 6.33 | 479.80 | 1376.11 | 699.81 | 8 | 19 | 7 |
| IPI00829877.1 | IGL IGL protein | 24.78 | 5.28 | 588.88 | 979.70 | 1329.90 | 8 | 14 | 12 |
| IPI00854806.1 | IGKV1 5 IGKV1 5 protein | 26.01 | 5.90 | 1034.30 | 1279.69 | 618.42 | 8 | 13 | 6 |
| IPI00878953.1 | APOE MRNA for apolipoprotein E | 32.54 | 7.10 | 608.11 | 692.88 | 661.23 | 8 | 12 | 12 |
| IPI00718819.2 | IGL IGLC2 IGLV2 14 IGLC1 IGLC3 IGLV2 14 | 24.68 | 5.87 | 747.31 | 892.57 | 652.68 | 8 | 11 | 12 |
| IPI00477090.6 | IGHM IGHM protein | 67.25 | 5.84 | 411.05 | 915.39 | 940.93 | 8 | 10 | 10 |
| IPI00896413.1 | ITIH4 100 kDa protein | 99.80 | 6.02 | 715.73 | 860.61 | 625.62 | 8 | 10 | 9 |
| IPI00385264.1 | Ig mu heavy chain disease protein | 43.03 | 4.95 | 491.95 | 473.36 | 265.60 | 8 | 8 | 4 |
| IPI00922043.1 | ITIH4 Protein | 84.19 | 5.16 | 1114.04 | 1349.50 | 1446.57 | 7 | 29 | 19 |
| IPI00789376.1 | KNG1 KNG1 protein | 33.06 | 6.26 | 253.77 | 1098.15 | 1328.57 | 7 | 19 | 26 |
| IPI00827488.1 | IGKC IGKC protein | 25.73 | 7.62 | 848.68 | 1540.95 | 279.56 | 7 | 11 | 5 |
| Tabl2 S1 Continued | |  |  |  |  |  |  |  |  |
| **Accession No.** | **Description** | **mW(kDa)** | **pI (pH)** | **PLGS Score** | | | **Peptides** | | |
|  |  |  |  | **C** | **IgAV** | **IgAVN** | **C** | **IgAV** | **IgAVN** |
| IPI00884926.1 | ORM1 orosomucoid 1 precursor | 23.52 | 4.83 | 366.59 | 142.87 | 951.10 | 9 | 6 | 5 |
| IPI00556287.1 | Putative uncharacterized protein | 20655 | 4.929 | 901.1306 | 977.6541 | 602.2183 | 7 | 9 | 6 |
| IPI00793626.1 | CP 22 kDa protein | 22.06 | 4.78 | 359.79 | 259.67 | 206.77 | 7 | 2 | 2 |
| IPI00796636.14 | HBB Hemoglobin Fragment | 11.50 | 5.90 | 349.40 | 1120.29 | 609.62 | 6 | 17 | 10 |
| IPI00784758.1 | LOC100126583 Putative uncharacterized protein DKFZp686M08189 | 52.20 | 4.97 | 764.93 | 1656.14 | 1094.73 | 6 | 16 | 7 |
| IPI00816555.1 | IGLC2 IGLV2 14 IGLC1 IGLC3 IGLV2 14 protein | 24.49 | 7.09 | 580.80 | 748.18 | 451.04 | 6 | 9 | 9 |
| IPI00784865.1 | IGK IGK protein | 25.76 | 5.91 | 844.86 | 932.17 | 675.06 | 6 | 8 | 6 |
| IPI00791330.1 | TF 8 kDa protein | 7.68 | 7.93 | 655.54 | 507.29 | 659.24 | 6 | 5 | 5 |
| IPI00892870.1 | IGHM Protein | 51.47 | 6.01 | 684.05 | 406.06 | 941.63 | 6 | 3 | 11 |
| IPI00816118.1 | IGKC IGKC protein | 25.91 | 8.16 | 816.74 | 1486.48 | 738.86 | 5 | 10 | 7 |
| IPI00550162.2 | IGLV3 25 IGLV3 25 protein | 24.85 | 5.05 | 262.79 | 851.72 | 369.09 | 5 | 7 | 6 |
| IPI00908365.1 | cDNA FLJ52255 highly similar to Angiotensinogen | 44.38 | 5.58 | 518.21 | 304.01 | 296.95 | 5 | 5 | 2 |
| IPI00022417.4 | LRG1 Leucine rich alpha 2 glycoprotein | 38.15 | 6.50 | 150.02 | 149.25 | 163.68 | 5 | 4 | 4 |
| IPI00893853.1 | IGHA2 Protein | 30.28 | 5.46 | 434.27 | 247.64 | 441.80 | 5 | 4 | 7 |
| IPI00853068.1 | HBA2 HBA1 Alpha 2 globin variant Fragment | 15.27 | 9.18 | 531.43 | 138.66 | 370.98 | 5 | 3 | 7 |
| IPI00879084.1 | CP 20 kDa protein | 20.02 | 7.57 | 283.26 | 178.70 | 602.17 | 5 | 2 | 9 |
| IPI00896419.2 | ITIH4 ITIH4 protein | 103.82 | 6.44 | 717.97 | 839.62 | 1518.51 | 4 | 15 | 25 |
| IPI00423461.3 | IGHA2 Putative uncharacterized protein DKFZp686C02220 Fragment | 54.13 | 5.88 | 502.08 | 878.86 | 365.90 | 4 | 14 | 4 |
| IPI00830047.1 | Putative uncharacterized protein ENSP00000374858 Fragment | 11.29 | 7.07 | 524.60 | 906.81 | 1102.20 | 4 | 14 | 12 |
| Tabl2 S1 Continued | |  |  |  |  |  |  |  |  |
| **Accession No.** | **Description** | **mW(kDa)** | **pI (pH)** | **PLGS Score** | | | **Peptides** | | |
|  |  |  |  | **C** | **IgAV** | **IgAVN** | **C** | **IgAV** | **IgAVN** |
| IPI00719373.2 | IGL IGL protein | 23.05 | 9.02 | 414.35 | 1041.73 | 144.24 | 4 | 14 | 3 |
| IPI00032220.3 | AGT Angiotensinogen | 53.12 | 5.85 | 1210.17 | 1086.63 | 620.02 | 4 | 13 | 15 |
| IPI00450309.1 | IGL IGL protein | 24.87 | 6.32 | 393.44 | 935.22 | 270.17 | 4 | 9 | 2 |
| IPI00784935.1 | IGL IGL protein | 24.64 | 5.92 | 391.76 | 277.95 | 993.05 | 4 | 4 | 9 |
| IPI00022429.3 | ORM1 Alpha 1 acid glycoprotein 1 | 23.50 | 4.74 | 366.59 | 464.51 | 951.10 | 4 | 3 | 11 |
| IPI00657660.1 | HBD 15 kDa protein | 15.41 | 8.00 | 364.21 | 151.44 | 408.70 | 4 | 1 | 7 |
| IPI00807459.1 | IGKC IGKC protein | 32.10 | 6.66 | 390.42 | 1506.86 | 684.65 | 3 | 9 | 7 |
| IPI00852577.2 | C1 segment protein | 11.39 | 7.99 | 214.90 | 580.46 | 216.43 | 3 | 9 | 3 |
| IPI00472961.2 | IGKC IGKC protein | 25.92 | 8.49 | 376.47 | 1038.60 | 655.83 | 3 | 6 | 7 |
| IPI00642632.1 | C7 protein | 11.35 | 8.48 | 272.72 | 537.35 | 757.36 | 3 | 6 | 11 |
| IPI00853045.1 | IGKC Anti RhD monoclonal T125 kappa light chain | 25.68 | 8.48 | 385.85 | 596.54 | 1665.90 | 3 | 5 | 18 |
| IPI00816799.1 | Rheumatoid factor D5 light chain | 12.76 | 9.28 | 486.33 | 665.32 | 126.40 | 3 | 4 | 2 |
| IPI00657911.2 | HBG2 Gamma globin | 15.31 | 9.92 | 160.58 | 146.12 | 156.62 | 3 | 3 | 3 |
| IPI00890703.1 | Cryocrystalglobulin CC1 kappa light chain variable region Fragment | 11788 | 8.214 | 178.8164 | 308.5941 | 128.4129 | 3 | 3 | 1 |
| IPI00478600.3 | IGKV1 5 IGKV1 5 protein | 25854 | 6.929 | 421.2781 | 585.7326 | 385.5063 | 3 | 2 | 4 |
| IPI00658130.1 | IGL IGL protein | 25.01 | 7.98 | 333.72 | 1256.64 | 808.11 | 2 | 15 | 11 |
| IPI00022432.1 | TTR Transthyretin | 15.88 | 5.40 | 153.21 | 737.29 | 99.50 | 2 | 7 | 2 |
| IPI00745660.2 | IGL IGL protein | 24.84 | 7.71 | 225.41 | 800.18 | 100.00 | 2 | 7 | 3 |
| IPI00798430.2 | TF 17 kDa protein | 17.22 | 5.23 | 129.14 | 763.13 | 1176.03 | 2 | 7 | 7 |
| IPI00657670.1 | Apolipoprotein C III variant 1 | 12.81 | 8.72 | 699.19 | 925.38 | 759.11 | 2 | 6 | 5 |
| IPI00178926.2 | IGJ immunoglobulin J chain | 18.09 | 4.91 | 168.39 | 97.56 | 115.27 | 2 | 4 | 1 |
| Tabl2 S1 Continued | |  |  |  |  |  |  |  |  |
| **Accession No.** | **Description** | **mW(kDa)** | **pI (pH)** | **PLGS Score** | | | **Peptides** | | |
|  |  |  |  | **C** | **IgAV** | **IgAVN** | **C** | **IgAV** | **IgAVN** |
| IPI00186903.4 | APOL1 Isoform 2 of Apolipoprotein L1 | 45.89 | 5.87 | 112.03 | 225.68 | 178.82 | 2 | 3 | 2 |
| IPI00789295.1 | TF 11 kDa protein | 11154 | 4.591 | 238.6913 | 195.0998 | 281.3025 | 2 | 3 | 2 |
| IPI00827826.1 | Cold agglutinin FS 2 L chain Fragment | 11881 | 9.302 | 237.494 | 261.1927 | 204.7541 | 2 | 2 | 1 |
| IPI00853641.1 | HBE1 Putative uncharacterized protein HBE1 | 9463 | 9.719 | 135.5786 | 117.8198 | 228.8153 | 2 | 2 | 4 |
| IPI00761125.3 | IGKC IGKC protein | 25.66 | 8.36 | 371.29 | 1026.87 | 384.40 | 1 | 7 | 2 |
| IPI00746963.1 | IGKC IGKC protein | 25.59 | 7.63 | 371.29 | 1023.36 | 269.00 | 1 | 5 | 2 |
| IPI00829711.1 | IGHA2 Putative uncharacterized protein IGHA2 Fragment | 42.35 | 5.29 | 211.95 | 382.43 | 158.46 | 1 | 5 | 2 |
| IPI00879456.1 | APOE 25 kDa protein | 24888 | 5.598 | 138.2249 | 176.6558 | 126.6676 | 1 | 3 | 1 |
| IPI00382436.1 | Ig lambda chain V III region SH | 11385 | 6.067 | 78.6736 | 149.0224 | 107.731 | 1 | 2 | 1 |
| IPI00923480.1 | Anti ED B scFV Fragment | 25189 | 8.216 | 278.5174 | 183.054 | 107.9167 | 1 | 2 | 3 |
| IPI00382440.1 | Ig lambda chain V IV region Hil | 11509 | 6.453 | 139.5853 | 174.9962 | 321.2531 | 1 | 1 | 2 |
| **Proteins shared between IgAV and IgAVN** | |  |  |  |  |  |  |  |  |
| IPI00829640.1 | IGL IGL protein | 24.84 | 6.33 |  | 1376.11 | 699.81 |  | 19 | 7 |
| IPI00797097.1 | KNG1 17 kDa protein | 17.34 | 4.63 |  | 718.21 | 249.91 |  | 14 | 3 |
| IPI00022395.1 | C9 Complement component C9 | 63.13 | 5.27 |  | 457.48 | 1334.26 |  | 12 | 38 |
| IPI00555945.1 | IGL IGL protein | 24.78 | 7.07 |  | 1038.86 | 244.99 |  | 12 | 4 |
| IPI00855916.1 | Transthyretin | 20.19 | 4.97 |  | 334.40 | 99.50 |  | 3 | 4 |
| IPI00827875.1 | Lambda chain | 24.64 | 7.57 |  | 861.89 | 169.29 |  | 9 | 2 |
| IPI00019399.1 | SAA4 Serum amyloid A 4 protein | 14.80 | 9.52 |  | 242.42 | 252.06 |  | 8 | 5 |
| IPI00513782.3 | GSN cDNA FLJ35478 fis clone SMINT2007796 highly similar to Gelsolin | 52.34 | 5.02 |  | 373.50 | 332.74 |  | 7 | 4 |
| IPI00896371.1 | IGHM Isoform 1 of Ig mu chain C region | 49.28 | 6.33 |  | 500.93 | 934.18 |  | 7 | 11 |
| Tabl2 S1 Continued | |  |  |  |  |  |  |  |  |
| **Accession No.** | **Description** | **mW(kDa)** | **pI (pH)** | **PLGS Score** | | | **Peptides** | | |
|  |  |  |  | **C** | **IgAV** | **IgAVN** | **C** | **IgAV** | **IgAVN** |
| IPI00419424.3 | IGKV1 5 IGKV1 5 protein | 26.22 | 6.34 |  | 1060.04 | 971.22 |  | 6 | 5 |
| IPI00796316.2 | GSN cDNA FLJ53327 highly similar to Gelsolin | 77.74 | 5.34 |  | 358.02 | 490.93 |  | 6 | 4 |
| IPI00298971.1 | VTN Vitronectin | 54.27 | 5.43 |  | 323.85 | 660.93 |  | 5 | 22 |
| IPI00789954.1 | TF 7 kDa protein | 7.29 | 6.08 |  | 406.38 | 320.82 |  | 5 | 1 |
| IPI00922564.1 | cDNA FLJ56652 highly similar to Hemopexin | 15.71 | 6.49 |  | 424.77 | 434.39 |  | 5 | 8 |
| IPI00829896.1 | HBD Hemoglobin Lepore Baltimore Fragment | 11.45 | 6.21 |  | 126.94 | 272.34 |  | 4 | 8 |
| IPI00887555.1 | LOC100133842 similar to lectin galactoside binding soluble 3 binding prot | 61.61 | 5.12 |  | 162.71 | 206.76 |  | 4 | 12 |
| IPI00896380.1 | IGHM Isoform 2 of Ig mu chain C region | 51.76 | 5.73 |  | 500.93 | 567.85 |  | 4 | 5 |
| IPI00218192.2 | ITIH4 Isoform 2 of Inter alpha trypsin inhibitor heavy chain H4 | 101.15 | 6.20 |  | 503.26 | 1591.03 |  | 3 | 14 |
| IPI00430820.1 | IGKV1 5 IGKV1 5 protein | 25748 | 5.649 |  | 481.6186 | 272.1376 |  | 3 | 3 |
| IPI00744476.6 | IGL IGL protein | 24808 | 6.333 |  | 273.0367 | 165.047 |  | 3 | 2 |
| IPI00796830.1 | A2M 13 kDa protein | 12984 | 6.107 |  | 172.6506 | 204.3163 |  | 3 | 3 |
| IPI00385252.1 | Ig kappa chain V III region GOL | 11822 | 9.502 |  | 287.8644 | 415.608 |  | 2 | 2 |
| IPI00430847.1 | IGKC IGKC protein | 25690 | 7.994 |  | 593.1921 | 265.1485 |  | 2 | 3 |
| IPI00793108.1 | CP 20 kDa protein | 20.18 | 5.15 |  | 112.69 | 257.40 |  | 2 | 4 |
| IPI00815947.1 | HBB Truncated beta globin Fragment | 4505 | 9.958 |  | 158.3987 | 70.0189 |  | 2 | 2 |
| IPI00922117.1 | cDNA FLJ51564 highly similar to Pregnancy zone protein | 14.96 | 4.90 |  | 118.98 | 259.72 |  | 2 | 7 |
| IPI00021857.1 | APOC3 Apolipoprotein C III | 10.85 | 5.05 |  | 253.25 | 567.25 |  | 1 | 8 |
| IPI00893178.1 | IGL 23 kDa protein | 23.14 | 9.02 |  | 237.01 | 390.05 |  | 1 | 4 |
| Tabl2 S1 Continued | |  |  |  |  |  |  |  |  |
| **Accession No.** | **Description** | **mW(kDa)** | **pI (pH)** | **PLGS Score** | | | **Peptides** | | |
|  |  |  |  | **C** | **IgAV** | **IgAVN** | **C** | **IgAV** | **IgAVN** |
| **Proteins shared between controls and IgAV** | |  |  |  |  |  |  |  |  |
| IPI00742696.2 | GC vitamin D binding protein precursor | 52.88 | 5.15 | 889.85 | 269.64 |  | 30 | 5 |  |
| IPI00555812.4 | GC Vitamin D binding protein | 52.93 | 5.24 | 864.50 | 271.52 |  | 22 | 10 |  |
| IPI00745089.2 | A1BG alpha 1B glycoprotein precursor | 54.22 | 5.48 | 1282.17 | 1049.89 |  | 17 | 12 |  |
| IPI00867588.1 | FN1 Isoform 13 of Fibronectin | 249.15 | 5.25 | 2165.21 | 2230.92 |  | 23 | 25 |  |
| IPI00472610.2 | IGHM IGHM protein | 52.63 | 7.41 | 736.66 | 527.76 |  | 12 | 10 |  |
| IPI00792626.1 | TF 14 kDa protein | 13.85 | 5.39 | 1349.93 | 1443.09 |  | 10 | 19 |  |
| IPI00793848.1 | CLU 54 kDa protein | 53.48 | 6.52 | 284.04 | 152.45 |  | 9 | 6 |  |
| IPI00291262.3 | CLU Clusterin | 52.46 | 5.84 | 694.15 | 172.28 |  | 6 | 3 |  |
| IPI00872895.1 | Putative uncharacterized protein ENSP00000381660 Fragment | 13415 | 8.763 | 271.0507 | 481.4805 |  | 5 | 6 |  |
| IPI00845354.1 | IGKC IGKC protein | 25.37 | 6.34 | 272.63 | 286.89 |  | 5 | 4 |  |
| IPI00784985.1 | IGK IGK protein | 25.50 | 6.14 | 818.99 | 603.68 |  | 5 | 2 |  |
| IPI00719452.1 | IGL IGL protein | 24.69 | 6.47 | 395.53 | 1174.56 |  | 3 | 9 |  |
| IPI00479116.1 | CPN2 Carboxypeptidase N subunit 2 | 60.58 | 5.57 | 262.70 | 208.80 |  | 3 | 4 |  |
| IPI00021856.3 | APOC2 Apolipoprotein C II | 11.28 | 4.44 | 117.28 | 136.99 |  | 1 | 2 |  |
| IPI00879937.1 | SERPINF2 28 kDa protein | 27805 | 6.325 | 152.8715 | 161.4308 |  | 2 | 3 |  |
| IPI00888118.1 | IGLC1 IGLC1 protein | 24786 | 5.892 | 152.2943 | 277.5957 |  | 2 | 2 |  |
| **Proteins shared between controls and IgAVN** | |  |  |  |  |  |  |  |  |
| IPI00339224.1 | FN1 Isoform 4 of Fibronectin | 222.80 | 5.30 | 2010.77 |  | 2059.00 | 22 |  | 32 |
| IPI00339226.1 | FN1 Isoform 6 of Fibronectin | 240.32 | 5.31 | 2124.12 |  | 1036.02 | 21 |  | 15 |
| IPI00884981.1 | PZP Isoform 2 of Pregnancy zone | 140.28 | 5.86 | 689.11 |  | 437.47 | 14 |  | 5 |
| IPI00748437.2 | PZP Putative uncharacterized protein PZP | 140.28 | 5.86 | 689.11 |  | 437.47 | 10 |  | 3 |
| Tabl2 S1 Continued | |  |  |  |  |  |  |  |  |
| **Accession No.** | **Description** | **mW(kDa)** | **pI (pH)** | **PLGS Score** | | | **Peptides** | | |
|  |  |  |  | **C** | **IgAV** | **IgAVN** | **C** | **IgAV** | **IgAVN** |
| IPI00646799.1 | A1BG cDNA FLJ31323 fis clone LIVER2000037 highly similar to Alpha 1B glyco | 35.25 | 5.94 | 596.12 |  | 424.02 | 12 |  | 6 |
| IPI00902880.1 | cDNA FLJ42722 fis clone BRAMY4000277 highly similar to Alpha 1B glycopro | 36.98 | 5.27 | 390.67 |  | 229.54 | 10 |  | 5 |
| IPI00791558.1 | HBD 11 kDa protein | 11.34 | 5.89 | 397.12 |  | 152.18 | 7 |  | 2 |
| IPI00878729.1 | A2M 19 kDa protein | 18.71 | 6.09 | 791.78 |  | 621.68 | 6 |  | 4 |
| IPI00025426.2 | PZP Isoform 1 of Pregnancy zone protein | 163.73 | 5.93 | 719.08 |  | 494.93 | 7 |  | 5 |
| IPI00514475.5 | APOL1 Isoform 1 of Apolipoprotein L1 | 43.95 | 5.47 | 106.02 |  | 170.72 | 5 |  | 5 |
| IPI00647556.2 | GSN gelsolin isoform c | 81.89 | 5.47 | 418.84 |  | 515.33 | 4 |  | 4 |
| IPI00807428.1 | Putative uncharacterized protein | 24731 | 5.568 | 300.1176 |  | 301.401 | 3 |  | 3 |
| IPI00220706.10 | HBG1 Hemoglobin subunit gamma 1 | 16.13 | 6.78 | 162.40 |  | 161.39 | 2 |  | 4 |
| IPI00394992.1 | PGLYRP2 Isoform 2 of N acetylmuramoyl L alanine amidase | 67.96 | 7.47 | 224.72 |  | 284.38 | 2 |  | 3 |
| IPI00029863.4 | 55 kDa protein | 55029 | 5.952 | 207.4086 |  | 125.6098 | 1 |  | 1 |
| **Proteins only in controls** | |  |  |  |  |  |  |  |  |
| IPI00017891.3 | APC2 Isoform 2 of Adenomatous polyposis coli protein 2 | 213.67 | 9.68 | 529.71 |  |  | 29 |  |  |
| IPI00382606.1 | F7 Factor VII mutant immunoconjugate | 75.50 | 6.59 | 1050.42 |  |  | 19 |  |  |
| IPI00448938.1 | IGHG1 IGHG1 protein | 51.36 | 8.44 | 978.12 |  |  | 18 |  |  |
| IPI00411462.7 | FN1 Isoform 2 of Fibronectin | 71.90 | 6.54 | 314.85 |  |  | 17 |  |  |
| IPI00423463.1 | IGHG1 Putative uncharacterized protein DKFZp686O01196 | 52.58 | 8.14 | 1011.46 |  |  | 17 |  |  |
| IPI00784810.1 | IGHV4 31 IGHV4 31 protein | 51.17 | 8.13 | 952.34 |  |  | 16 |  |  |
| Tabl2 S1 Continued | |  |  |  |  |  |  |  |  |
| **Accession No.** | **Description** | **mW(kDa)** | **pI (pH)** | **PLGS Score** | | | **Peptides** | | |
|  |  |  |  | **C** | **IgAV** | **IgAVN** | **C** | **IgAV** | **IgAVN** |
| IPI00175193.5 | KIF4B Chromosome associated kinesin KIF4B | 139.95 | 5.79 | 351.96 |  |  | 15 |  |  |
| IPI00219561.1 | NLRP14 NACHT LRR and PYD domains containing protein 14 | 124.65 | 6.16 | 443.78 |  |  | 15 |  |  |
| IPI00448925.3 | IGHG1 IGHG1 protein | 60.06 | 7.36 | 986.29 |  |  | 15 |  |  |
| IPI00645363.2 | IGHG1 Putative uncharacterized protein DKFZp686P15220 | 51.69 | 7.84 | 993.88 |  |  | 15 |  |  |
| IPI00290283.6 | MASP1 mannan binding lectin serine protease 1 isoform 2 precursor | 81.81 | 4.82 | 258.21 |  |  | 12 |  |  |
| IPI00384938.1 | IGHG1 Putative uncharacterized protein DKFZp686N02209 | 52.82 | 8.49 | 952.74 |  |  | 12 |  |  |
| IPI00784842.1 | IGHV4 31 Putative uncharacterized protein DKFZp686G11190 | 52.01 | 7.99 | 960.07 |  |  | 12 |  |  |
| IPI00816314.1 | IGHM Putative uncharacterized protein DKFZp686I15196 | 50.89 | 8.00 | 868.21 |  |  | 12 |  |  |
| IPI00816681.1 | IGHM Hepatitis B virus receptor binding protein Fragment | 38.14 | 7.99 | 1009.59 |  |  | 12 |  |  |
| IPI00178150.5 | KIF4A Isoform 1 of Chromosome associated kinesin KIF4A | 139.79 | 5.89 | 280.71 |  |  | 11 |  |  |
| IPI00556059.1 | KIF4A Isoform 2 of Chromosome associated kinesin KIF4A | 128.38 | 5.81 | 262.20 |  |  | 11 |  |  |
| IPI00423466.1 | IGHG1 Putative uncharacterized protein DKFZp686H20196 | 52.73 | 8.49 | 1020.51 |  |  | 11 |  |  |
| IPI00807531.2 | IGHG1 IGHG1 protein | 51.95 | 8.22 | 948.29 |  |  | 11 |  |  |
| Tabl2 S1 Continued | |  |  |  |  |  |  |  |  |
| **Accession No.** | **Description** | **mW(kDa)** | **pI (pH)** | **PLGS Score** | | | **Peptides** | | |
|  |  |  |  | **C** | **IgAV** | **IgAVN** | **C** | **IgAV** | **IgAVN** |
| IPI00761159.1 | IGHM IGHM protein | 52.55 | 7.45 | 838.62 |  |  | 10 |  |  |
| IPI00785084.1 | IGHV4 31 Immunoglobulin heavy variable 4 31 | 52.25 | 8.67 | 720.19 |  |  | 10 |  |  |
| IPI00910714.1 | cDNA FLJ54754 highly similar to Protein FAM61A | 46.38 | 9.87 | 137.04 |  |  | 10 |  |  |
| IPI00022431.2 | AHSG cDNA FLJ55606 highly similar to Alpha 2 HS glycoprotein | 46.60 | 5.82 | 206.91 |  |  | 9 |  |  |
| IPI00914853.1 | LRRFIP1 leucine rich repeat in FLII interacting protein 1 isoform 2 | 44.88 | 5.34 | 196.62 |  |  | 9 |  |  |
| IPI00299906.4 | HIF1AN cDNA FLJ60091 highly similar to Hypoxia inducible factor 1 alpha inh | 44.25 | 5.78 | 241.04 |  |  | 8 |  |  |
| IPI00641877.1 | WNT2B Protein Wnt | 33.92 | 8.88 | 82.65 |  |  | 8 |  |  |
| IPI00829944.1 | IGHG1 IGHG1 protein | 51.22 | 7.65 | 561.52 |  |  | 8 |  |  |
| IPI00871960.2 | MASP1 cDNA FLJ75203 highly similar to Homo sapiens mannan binding lectin se | 81.74 | 4.85 | 258.21 |  |  | 8 |  |  |
| IPI00880060.1 | GSK3A Putative uncharacterized protein GSK3A | 44.89 | 8.17 | 102.50 |  |  | 8 |  |  |
| IPI00423464.1 | IGHG1 Putative uncharacterized protein DKFZp686K03196 | 52.33 | 8.61 | 475.00 |  |  | 7 |  |  |
| IPI00550640.2 | IGHG4 IGHG4 protein | 51.95 | 7.84 | 550.43 |  |  | 7 |  |  |
| IPI00888398.2 | RNF187 Protein RNF187 | 14.61 | 5.82 | 139.03 |  |  | 7 |  |  |
| IPI00784817.1 | IGHV4 31 Anti RhD monoclonal T125 gamma1 heavy chain | 52.33 | 8.32 | 722.05 |  |  | 7 |  |  |
| IPI00876888.1 | cDNA FLJ78387 | 51.56 | 8.13 | 760.01 |  |  | 7 |  |  |
| IPI00795830.1 | AHSG 29 kDa protein | 28.52 | 4.55 | 174.02 |  |  | 7 |  |  |
| Tabl2 S1 Continued | |  |  |  |  |  |  |  |  |
| **Accession No.** | **Description** | **mW(kDa)** | **pI (pH)** | **PLGS Score** | | | **Peptides** | | |
|  |  |  |  | **C** | **IgAV** | **IgAVN** | **C** | **IgAV** | **IgAVN** |
| IPI00005686.1 | LIPG Isoform 1 of Endothelial lipase | 56.76 | 7.82 | 211.33 |  |  | 6 |  |  |
| IPI00409639.1 | METTL13 Isoform 4 of Putative methyltransferase KIAA0859 | 61.09 | 5.71 | 247.35 |  |  | 6 |  |  |
| IPI00426051.3 | LOC100133739 Putative uncharacterized protein DKFZp686C15213 | 51.07 | 7.58 | 429.42 |  |  | 6 |  |  |
| IPI00784822.1 | IGHV4 31 IGHV4 31 protein | 51.31 | 7.46 | 227.57 |  |  | 6 |  |  |
| IPI00794403.1 | LUM 23 kDa protein | 23.14 | 8.94 | 258.61 |  |  | 6 |  |  |
| IPI00829814.1 | IGHG4 Ig gamma 4 chain C region | 35.92 | 7.11 | 518.46 |  |  | 6 |  |  |
| IPI00844156.2 | SERPINC1 SERPINC1 protein | 29.07 | 8.99 | 259.49 |  |  | 6 |  |  |
| IPI00013698.1 | ASAH1 Acid ceramidase | 44.62 | 7.52 | 101.87 |  |  | 5 |  |  |
| IPI00020986.2 | LUM Lumican | 38.40 | 6.16 | 256.79 |  |  | 5 |  |  |
| IPI00399007.5 | IGHG2 Putative uncharacterized protein DKFZp686I04196 Fragment | 46.03 | 7.42 | 406.99 |  |  | 5 |  |  |
| IPI00830132.1 | IGHG4 Putative uncharacterized protein IGHG4 | 43.34 | 8.02 | 530.64 |  |  | 5 |  |  |
| IPI00910892.1 | cDNA FLJ51979 highly similar to Tumor necrosis factor receptor superfamil | 19.21 | 7.88 | 136.41 |  |  | 5 |  |  |
| IPI00002919.2 | DIRAS1 GTP binding protein Di Ras1 | 22.31 | 8.94 | 133.72 |  |  | 4 |  |  |
| IPI00009815.1 | C1orf156 UPF0558 protein C1orf156 | 42.12 | 6.30 | 140.04 |  |  | 4 |  |  |
| IPI00023019.1 | SHBG Isoform 1 of Sex hormone binding globulin | 43.75 | 6.24 | 133.43 |  |  | 4 |  |  |
| IPI00103702.1 | C10orf54 Putative uncharacterized protein PP2135 | 19.03 | 9.95 | 118.75 |  |  | 4 |  |  |
| IPI00418446.4 | ASAH1 N acylsphingosine amidohydrolase 1 isoform b | 46.47 | 7.82 | 94.72 |  |  | 4 |  |  |
| Tabl2 S1 Continued | |  |  |  |  |  |  |  |  |
| **Accession No.** | **Description** | **mW(kDa)** | **pI (pH)** | **PLGS Score** | | | **Peptides** | | |
|  |  |  |  | **C** | **IgAV** | **IgAVN** | **C** | **IgAV** | **IgAVN** |
| IPI00555799.1 | HSP90AA6P Heat shock protein 90Af | 11.74 | 8.79 | 62.58 |  |  | 4 |  |  |
| IPI00922262.1 | cDNA FLJ56822 highly similar to Alpha 2 HS glycoprotein | 27.34 | 6.07 | 145.27 |  |  | 4 |  |  |
| IPI00001794.2 | INSIG2 Insulin induced gene 2 | 24761 | 7.919 | 98.4563 |  |  | 3 |  |  |
| IPI00384061.2 | METTL13 Isoform 5 of Putative methyltransferase KIAA0859 | 44.53 | 5.29 | 154.87 |  |  | 3 |  |  |
| IPI00411817.2 | ACCS 1 aminocyclopropane 1 carboxylate synthase like protein 1 | 57.29 | 5.98 | 154.89 |  |  | 3 |  |  |
| IPI00744618.2 | STMN1 Stathmin | 12.81 | 8.79 | 140.73 |  |  | 3 |  |  |
| IPI00745231.2 | WEE2 Wee1 like protein kinase 2 | 62.88 | 6.01 | 127.58 |  |  | 3 |  |  |
| IPI00797570.1 | Hypothetical short protein | 7.28 | 5.34 | 59.45 |  |  | 3 |  |  |
| IPI00885193.1 | IGKV3 20 Myosin reactive immunoglobulin light chain variable region Fragmen | 11.64 | 8.47 | 531.28 |  |  | 3 |  |  |
| IPI00885193.1 | IGKV3 20 Myosin reactive immunoglobulin light chain variable region Fragmen | 11.64 | 8.47 | 531.28 |  |  | 3 |  |  |
| IPI00163207.1 | PGLYRP2 Isoform 1 of N acetylmuramoyl L alanine amidase | 62.18 | 7.27 | 215.28 |  |  | 2 |  |  |
| IPI00221187.1 | NOVA1 Isoform 2 of RNA binding protein Nova 1 | 19.48 | 9.65 | 111.13 |  |  | 2 |  |  |
| IPI00386839.1 | Amyloid lambda 6 light chain variable region SAR Fragment | 12.29 | 4.63 | 123.60 |  |  | 2 |  |  |
| IPI00410590.5 | LSM14A Isoform 2 of Protein LSM14 homolog A | 50.52 | 9.84 | 122.20 |  |  | 2 |  |  |
| IPI00554676.2 | HBG2 HBG1 Hemoglobin subunit gamma 2 | 16.12 | 6.78 | 164.49 |  |  | 2 |  |  |
| IPI00892671.1 | IGHG1 32 kDa protein | 31.96 | 8.00 | 197.57 |  |  | 2 |  |  |
|  |  |  |  |  |  |  |  |  |  |
| Table S2 Continued | |  |  |  |  |  |  |  |  |
| **Accession No.** | **Description** | **mW(kDa)** | **pI (pH)** | **PLGS Score** | | | **Peptides** | | |
|  |  |  |  | **C** | **IgAV** | **IgAVN** | **C** | **IgAV** | **IgAVN** |
| IPI00555554.1 | ITGB8 Integrin beta Fragment | 18.79 | 6.22 | 111.28 |  |  | 1 |  |  |
| IPI00217471.3 | HBE1 Hemoglobin subunit epsilon | 16.19 | 9.18 | 152.21 |  |  | 1 |  |  |
| IPI00736860.3 | Protein | 36.27 | 7.74 | 138.88 |  |  | 1 |  |  |
| **Proteins only in IgAV** | |  |  |  |  |  |  |  |  |
| IPI00001849.5 | KIAA1530 Isoform 1 of Uncharacterized protein KIAA1530 | 80.56 | 5.88 |  | 292.36 |  |  | 15 |  |
| IPI00844578.1 | DHX9 ATP dependent RNA helicase A | 140.87 | 6.39 |  | 338.22 |  |  | 14 |  |
| IPI00012505.5 | TMPRSS13 Isoform 3 of Transmembrane protease serine 13 | 57.59 | 8.55 |  | 213.48 |  |  | 12 |  |
| IPI00783287.1 | Immunglobulin heavy chain variable region Fragment | 13.43 | 8.74 |  | 741.31 |  |  | 10 |  |
| IPI00453467.7 | C1orf173 Isoform 3 of Uncharacterized protein | 60.22 | 5.71 |  | 190.65 |  |  | 9 |  |
| IPI00549916.2 | UBXN11 Isoform 7 of UBX domain containing protein 11 | 32.88 | 4.78 |  | 137.62 |  |  | 9 |  |
| IPI00783689.1 | Immunglobulin heavy chain variable region Fragment | 12.97 | 8.19 |  | 247.25 |  |  | 9 |  |
| IPI00015175.1 | WNT2 Protein Wnt 2 | 40.39 | 8.65 |  | 235.70 |  |  | 8 |  |
| IPI00019580.1 | PLG Plasminogen | 90.51 | 6.91 |  | 242.39 |  |  | 8 |  |
| IPI00477357.3 | PLD5 Isoform 3 of Inactive phospholipase D5 | 37.71 | 9.56 |  | 234.25 |  |  | 8 |  |
| IPI00158144.4 | SYCE2 Synaptonemal complex central element 2 | 24.67 | 5.47 |  | 167.73 |  |  | 6 |  |
| IPI00783451.1 | Immunglobulin heavy chain variable region | 13.61 | 8.18 |  | 257.60 |  |  | 6 |  |
| IPI00218732.3 | PON1 Serum paraoxonase arylesterase 1 | 39.72 | 4.92 |  | 193.95 |  |  | 5 |  |
| Table S2 Continued | |  |  |  |  |  |  |  |  |
| **Accession No.** | **Description** | **mW(kDa)** | **pI (pH)** | **PLGS Score** | | | **Peptides** | | |
|  |  |  |  | **C** | **IgAV** | **IgAVN** | **C** | **IgAV** | **IgAVN** |
| IPI00552578.2 | SAA1 SAA2 Serum amyloid A protein | 13.52 | 6.35 |  | 97.14 |  |  | 5 |  |
| IPI00816007.1 | Putative uncharacterized protein | 17.53 | 12.10 |  | 98.45 |  |  | 5 |  |
| IPI00015388.1 | PAFAH2 Platelet activating factor acetylhydrolase 2 cytoplasmic | 44.01 | 6.44 |  | 142.13 |  |  | 4 |  |
| IPI00022995.1 | LOC100130401 PRO0641 | 6.14 | 9.82 |  | 69.73 |  |  | 4 |  |
| IPI00290444.2 | PPP1R15A Protein phosphatase 1 regulatory subunit 15A | 73.43 | 4.36 |  | 199.06 |  |  | 4 |  |
| IPI00382500.1 | Ig heavy chain V III region GAL | 12.72 | 8.81 |  | 109.26 |  |  | 4 |  |
| IPI00385985.1 | Ig lambda chain V III region LOI | 11.93 | 4.76 |  | 183.53 |  |  | 4 |  |
| IPI00455971.3 | MAGEA13P Putative MAGE domain containing protein ENSP00000355344 | 37.87 | 5.93 |  | 127.11 |  |  | 4 |  |
| IPI00815938.1 | IGLV3 21 IGLV3 21 protein | 24.88 | 6.58 |  | 480.68 |  |  | 4 |  |
| IPI00847136.1 | PLD5 Isoform 2 of Inactive phospholipase D5 | 50.76 | 9.52 |  | 246.19 |  |  | 4 |  |
| IPI00914948.1 | APOL1 apolipoprotein L1 isoform c precursor | 42.13 | 5.46 |  | 218.47 |  |  | 4 |  |
| IPI00914985.1 | Epididymis luminal protein 180 Fragment | 13.28 | 5.03 |  | 137.08 |  |  | 4 |  |
| IPI00167854.1 | LOC100132116 cDNA FLJ36021 fis clone TESTI2016568 | 14.69 | 8.45 |  | 89.80 |  |  | 3 |  |
| IPI00793155.2 | RAB34 RAB39 isoform 5 | 35.16 | 8.70 |  | 118.46 |  |  | 3 |  |
| IPI00304692.1 | RBMX Heterogeneous nuclear ribonucleoprotein G | 42.31 | 10.24 |  | 208.51 |  |  | 3 |  |
| IPI00382491.1 | Ig heavy chain V III region POM | 12945 | 8.201 |  | 121.1083 |  |  | 3 |  |
| IPI00384392.1 | Myosin reactive immunoglobulin heavy chain variable region Fragment | 12.86 | 6.54 |  | 320.88 |  |  | 3 |  |
| Table S2 Continued | |  |  |  |  |  |  |  |  |
| **Accession No.** | **Description** | **mW(kDa)** | **pI (pH)** | **PLGS Score** | | | **Peptides** | | |
|  |  |  |  | **C** | **IgAV** | **IgAVN** | **C** | **IgAV** | **IgAVN** |
| IPI00382497.1 | Ig heavy chain V III region TUR | 12423 | 10.03 |  | 108.063 |  |  | 3 |  |
| IPI00384401.1 | Myosin reactive immunoglobulin kappa chain variable region Fragment | 11.75 | 8.85 |  | 243.22 |  |  | 3 |  |
| IPI00387118.1 | Ig kappa chain V III region WOL | 11.74 | 9.21 |  | 312.03 |  |  | 3 |  |
| IPI00478105.4 | USH1C Isoform 4 of Harmonin | 60.29 | 5.10 |  | 147.61 |  |  | 3 |  |
| IPI00795399.1 | RAB34 25 kDa protein | 25.15 | 7.70 |  | 94.01 |  |  | 3 |  |
| IPI00847600.1 | PLD5 Isoform 1 of Inactive phospholipase D5 | 61.27 | 8.82 |  | 264.99 |  |  | 3 |  |
| IPI00872537.1 | USH1C 60 kDa protein | 60.44 | 5.06 |  | 163.93 |  |  | 3 |  |
| IPI00895943.1 | SAA1 SAA2 serum amyloid A2 isoform b | 9.18 | 7.16 |  | 110.21 |  |  | 3 |  |
| IPI00908406.1 | cDNA FLJ54253 highly similar to Ras related protein Rab 34 | 26.70 | 7.10 |  | 112.75 |  |  | 3 |  |
| IPI00910900.1 | cDNA FLJ51329 highly similar to Harmonin | 54.38 | 4.90 |  | 131.89 |  |  | 3 |  |
| IPI00000848.4 | TMPRSS13 Isoform 1 of Transmembrane protease serine 13 | 62640 | 8.5 |  | 216.8952 |  |  | 2 |  |
| IPI00007899.4 | Single chain Fv Fragment | 12234 | 8.76 |  | 103.8981 |  |  | 2 |  |
| IPI00167254.3 | PLD5 Isoform 4 of Inactive phospholipase D5 | 53.96 | 8.53 |  | 246.19 |  |  | 2 |  |
| IPI00382493.1 | Ig heavy chain V III region WAS | 13082 | 8.222 |  | 120.2443 |  |  | 2 |  |
| IPI00382494.1 | Ig heavy chain V III region TEI | 12794 | 8.799 |  | 165.7111 |  |  | 2 |  |
| IPI00387115.1 | Ig kappa chain V III region SIE | 11.77 | 8.79 |  | 315.35 |  |  | 2 |  |
| IPI00477860.2 | FAM38A Putative uncharacterized protein | 7.72 | 10.17 |  | 66.15 |  |  | 2 |  |
| IPI00744503.1 | HBG1 17 kDa protein | 17.30 | 6.05 |  | 153.35 |  |  | 2 |  |
| IPI00793653.1 | NCOR1 NCOR1 protein Fragment | 64.63 | 9.55 |  | 168.75 |  |  | 2 |  |
| IPI00794212.1 | NCOR1 NCOR1 protein | 23.70 | 8.58 |  | 134.52 |  |  | 2 |  |
| Table S2 Continued | |  |  |  |  |  |  |  |  |
| **Accession No.** | **Description** | **mW(kDa)** | **pI (pH)** | **PLGS Score** | | | **Peptides** | | |
|  |  |  |  | **C** | **IgAV** | **IgAVN** | **C** | **IgAV** | **IgAVN** |
| IPI00854667.1 | Putative uncharacterized protein ENSP00000375015 | 12.67 | 7.99 |  | 79.07 |  |  | 2 |  |
| IPI00872703.1 | TMPRSS13 transmembrane protease serine 13 | 61.46 | 8.66 |  | 212.34 |  |  | 2 |  |
| IPI00888708.1 | LOC100131704 similar to nuclear receptor co repressor 1 | 13.98 | 9.19 |  | 146.30 |  |  | 2 |  |
| IPI00027505.2 | ITGAV Isoform 1 of Integrin alpha V | 115.96 | 5.31 |  | 364.21 |  |  | 1 |  |
| IPI00385925.1 | TTC33 PRO2476 | 6.42 | 9.90 |  | 109.66 |  |  | 1 |  |
| IPI00387094.1 | Ig kappa chain V I region Hau | 11.66 | 8.79 |  | 79.46 |  |  | 1 |  |
| IPI00555872.5 | IGHV3 48 Myosin reactive immunoglobulin heavy chain variable region Fragmen | 12.84 | 5.04 |  | 302.32 |  |  | 1 |  |
| IPI00642023.2 | RDBP RD RNA binding protein | 17.28 | 10.10 |  | 75.76 |  |  | 1 |  |
| IPI00788698.1 | KNG1 6 kDa protein | 5.55 | 4.84 |  | 118.28 |  |  | 1 |  |
| IPI00916434.1 | Anti ED B scFV Fragment | 25.13 | 8.21 |  | 158.68 |  |  | 1 |  |
| **Proteins only in IgAVN** | |  |  |  |  |  |  |  |  |
| IPI00296421.2 | EHBP1L1 EH domain binding protein 1 like protein 1 | 161.76 | 4.60 |  |  | 339.89 |  |  | 23 |
| IPI00221112.3 | KIF9 cDNA FLJ57013 highly similar to Kinesin like protein KIF9 | 74.91 | 7.49 |  |  | 289.69 |  |  | 10 |
| IPI00829853.3 | AKAP13 Isoform 6 of A kinase anchor protein 13 | 49.02 | 9.19 |  |  | 148.13 |  |  | 10 |
| IPI00026314.1 | GSN Isoform 1 of Gelsolin | 85.64 | 5.84 |  |  | 361.77 |  |  | 9 |
| IPI00384479.7 | C12orf40 Isoform 2 of Uncharacterized protein | 54.85 | 8.76 |  |  | 151.12 |  |  | 9 |
| IPI00020091.1 | ORM2 Alpha 1 acid glycoprotein 2 | 23.59 | 4.85 |  |  | 269.93 |  |  | 8 |
| IPI00913983.1 | SYN3 synapsin III isoform IIIg | 63.18 | 9.70 |  |  | 220.03 |  |  | 8 |
| Table S2 Continued | |  |  |  |  |  |  |  |  |
| **Accession No.** | **Description** | **mW(kDa)** | **pI (pH)** | **PLGS Score** | | | **Peptides** | | |
|  |  |  |  | **C** | **IgAV** | **IgAVN** | **C** | **IgAV** | **IgAVN** |
| IPI00004527.2 | KIAA0355 Uncharacterized protein KIAA0355 | 115.95 | 6.72 |  |  | 198.09 |  |  | 7 |
| IPI00023673.1 | LGALS3BP Galectin 3 binding protein | 65.29 | 4.94 |  |  | 196.80 |  |  | 6 |
| IPI00218074.1 | FAM9C Protein FAM9C | 19.20 | 4.96 |  |  | 92.67 |  |  | 6 |
| IPI00876950.1 | ITIH3 Isoform 2 of Inter alpha trypsin inhibitor heavy chain H3 | 99.27 | 5.42 |  |  | 339.90 |  |  | 6 |
| IPI00031074.1 | ELAVL3 Putative uncharacterized protein DKFZp547J036 | 25.63 | 10.33 |  |  | 191.78 |  |  | 5 |
| IPI00644018.1 | A1BG 41 kDa protein | 40.69 | 5.40 |  |  | 511.56 |  |  | 5 |
| IPI00873416.1 | ITIH3 Putative uncharacterized protein ITIH3 | 75.03 | 5.49 |  |  | 288.37 |  |  | 5 |
| IPI00922076.1 | cDNA FLJ52288 highly similar to Armadillo repeat containing protein 4 | 23.29 | 8.80 |  |  | 133.57 |  |  | 5 |
| IPI00003951.7 | LAMA3 15 kDa protein | 14.92 | 9.87 |  |  | 108.92 |  |  | 4 |
| IPI00011694.1 | PRSS1 Trypsin 1 | 26.54 | 6.07 |  |  | 162.64 |  |  | 4 |
| IPI00029437.2 | KIF9 Isoform 1 of Kinesin like protein KIF9 | 89.96 | 6.61 |  |  | 305.86 |  |  | 4 |
| IPI00373789.2 | SYN3 synapsin III isoform IIIc | 48.96 | 8.92 |  |  | 214.15 |  |  | 4 |
| IPI00377087.4 | GSN Gelsolin | 20.77 | 4.49 |  |  | 126.74 |  |  | 4 |
| IPI00394924.1 | TCF23 Transcription factor 23 | 23.29 | 11.72 |  |  | 91.44 |  |  | 4 |
| IPI00449996.2 | HEPN1 Putative cancer susceptibility gene | 10.30 | 8.83 |  |  | 126.31 |  |  | 4 |
| IPI00646773.2 | GSN Isoform 2 of Gelsolin | 80.59 | 5.47 |  |  | 362.38 |  |  | 4 |
| IPI00745335.7 | GPX4 Isoform Cytoplasmic of Phospholipid hydroperoxide glutathione peroxidas | 19.47 | 7.85 |  |  | 237.24 |  |  | 4 |
| IPI00747654.1 | TTN CDNA FLJ26346 fis clone HRT04038 highly similar to Homo sapiens titin | 32.55 | 5.30 |  |  | 243.44 |  |  | 4 |
| Table S2 Continued | |  |  |  |  |  |  |  |  |
| **Accession No.** | **Description** | **mW(kDa)** | **pI (pH)** | **PLGS Score** | | | **Peptides** | | |
|  |  |  |  | **C** | **IgAV** | **IgAVN** | **C** | **IgAV** | **IgAVN** |
| IPI00797356.1 | CRBN 11 kDa protein | 10.74 | 9.23 |  |  | 111.18 |  |  | 4 |
| IPI00884192.1 | GPX4 glutathione peroxidase 4 isoform C precursor | 27.03 | 10.39 |  |  | 236.07 |  |  | 4 |
| IPI00909807.1 | cDNA FLJ60769 highly similar to Inter alpha trypsin inhibitor heavy chain | 78.07 | 6.01 |  |  | 329.36 |  |  | 4 |
| IPI00915024.1 | HBB Hemoglobin delta Etolia variant Fragment | 11.43 | 6.21 |  |  | 405.37 |  |  | 4 |
| IPI00921374.1 | LOC440386 Conserved hypothetical protein | 20.04 | 11.75 |  |  | 145.20 |  |  | 4 |
| IPI00006662.1 | APOD Apolipoprotein D | 21261 | 4.87 |  |  | 254.2098 |  |  | 3 |
| IPI00028413.8 | ITIH3 Isoform 1 of Inter alpha trypsin inhibitor heavy chain H3 | 99.79 | 5.38 |  |  | 345.46 |  |  | 3 |
| IPI00290198.3 | IL18 Interleukin 18 | 22.31 | 4.32 |  |  | 143.51 |  |  | 3 |
| IPI00304814.4 | GPX4 Isoform Mitochondrial of Phospholipid hydroperoxide glutathione peroxid | 22.11 | 8.29 |  |  | 236.07 |  |  | 3 |
| IPI00745800.1 | GPX4 glutathione peroxidase 4 isoform B precursor | 24.98 | 9.15 |  |  | 233.46 |  |  | 3 |
| IPI00788641.1 | POLG2 16 kDa protein | 15.61 | 6.36 |  |  | 163.97 |  |  | 3 |
| IPI00793596.1 | TESC 9 kDa protein | 9.01 | 4.38 |  |  | 66.29 |  |  | 3 |
| IPI00795582.2 | C12orf40 Isoform 1 of Uncharacterized protein C12orf40 | 74.46 | 7.82 |  |  | 155.00 |  |  | 3 |
| IPI00871622.2 | Putative zinc alpha 2 glycoprotein like 1 | 22.97 | 5.83 |  |  | 159.84 |  |  | 3 |
| IPI00908762.1 | cDNA FLJ53509 highly similar to Galectin 3 binding protein | 46.39 | 4.93 |  |  | 153.22 |  |  | 3 |
| IPI00021343.1 | IL1F9 Isoform 1 of Interleukin 1 family member 9 | 18.71 | 4.82 |  |  | 137.22 |  |  | 2 |
| Table S2 Continued | |  |  |  |  |  |  |  |  |
| **Accession No.** | **Description** | **mW(kDa)** | **pI (pH)** | **PLGS Score** | | | **Peptides** | | |
|  |  |  |  | **C** | **IgAV** | **IgAVN** | **C** | **IgAV** | **IgAVN** |
| IPI00298984.5 | SYN3 Synapsin 3 | 63.26 | 9.70 |  |  | 220.03 |  |  | 2 |
| IPI00452727.3 | HES6 Isoform 1 of Transcription cofactor HES 6 | 24.11 | 4.99 |  |  | 87.81 |  |  | 2 |
| IPI00452728.2 | HES6 Isoform 2 of Transcription cofactor HES 6 | 23.47 | 12.35 |  |  | 93.13 |  |  | 2 |
| IPI00742772.2 | Similar to Alpha 2 macroglobulin precursor | 8.47 | 8.00 |  |  | 125.91 |  |  | 2 |
| IPI00790430.1 | DPH3 7 kDa protein | 6.79 | 8.28 |  |  | 115.39 |  |  | 2 |
| IPI00792161.1 | ULK1 7 kDa protein | 7.11 | 7.37 |  |  | 140.84 |  |  | 2 |
| IPI00848182.1 | Conserved hypothetical protein | 10.58 | 9.92 |  |  | 123.78 |  |  | 2 |
| IPI00877152.1 | ZNF428 Isoform 2 of Zinc finger protein 428 | 13.06 | 3.91 |  |  | 76.08 |  |  | 2 |
| IPI00885201.1 | FAM172A Isoform 2 of UPF0528 protein FAM172A | 34.27 | 5.23 |  |  | 76.75 |  |  | 2 |
| IPI00914008.1 | MUC3B Conserved hypothetical protein | 37.67 | 9.49 |  |  | 205.23 |  |  | 2 |
| IPI00257450.9 | RGS22 Isoform 1 of Regulator of G protein signaling 22 | 147.07 | 7.80 |  |  | 291.29 |  |  | 1 |
| IPI00383795.1 | Mutant beta globin | 1.95 | 7.16 |  |  | 107.78 |  |  | 1 |
| IPI00792987.1 | UTS2D 9 kDa protein | 8.71 | 9.56 |  |  | 245.43 |  |  | 1 |
| IPI00828099.1 | UGa8H Fragment | 13.05 | 8.18 |  |  | 236.06 |  |  | 1 |
| IPI00877765.1 | APOL1 Apolipoprotein L 1 | 18.10 | 5.12 |  |  | 79.45 |  |  | 1 |
